# Neural network features distinguish chemosensory stimuli in *Caenorhabditis elegans*

**DOI:** 10.1371/journal.pcbi.1009591

**Published:** 2021-11-09

**Authors:** Javier J. How, Saket Navlakha, Sreekanth H. Chalasani

**Affiliations:** 1 Neurosciences Graduate Program, University of California, San Diego, La Jolla, California, United States of America; 2 Simons Center for Quantitative Biology, Cold Spring Harbor Laboratory, Cold Spring Harbor, New York, United States of America; 3 Molecular Neurobiology Laboratory, The Salk Institute for Biological Studies, San Diego, La Jolla, California, United States of America; Ernst-Strungmann-Institut, GERMANY

## Abstract

Nervous systems extract and process information from the environment to alter animal behavior and physiology. Despite progress in understanding how different stimuli are represented by changes in neuronal activity, less is known about how they affect broader neural network properties. We developed a framework for using graph-theoretic features of neural network activity to predict ecologically relevant stimulus properties, in particular stimulus identity. We used the transparent nematode, *Caenorhabditis elegans*, with its small nervous system to define neural network features associated with various chemosensory stimuli. We first immobilized animals using a microfluidic device and exposed their noses to chemical stimuli while monitoring changes in neural activity of more than 50 neurons in the head region. We found that graph-theoretic features, which capture patterns of interactions between neurons, are modulated by stimulus identity. Further, we show that a simple machine learning classifier trained using graph-theoretic features alone, or in combination with neural activity features, can accurately predict salt stimulus. Moreover, by focusing on putative causal interactions between neurons, the graph-theoretic features were almost twice as predictive as the neural activity features. These results reveal that stimulus identity modulates the broad, network-level organization of the nervous system, and that graph theory can be used to characterize these changes.

## Author summary

Animals use their nervous systems to detect and respond to changes in their external environment. A central challenge in computational neuroscience is to determine how specific properties of these stimuli affect interactions between neurons. While most studies have focused on the neurons in the sensory periphery, recent advances allow us to probe how the rest of the nervous system responds to sensory stimulation. We recorded activity of neurons within the *C*. *elegans* head region while the animal was exposed to various chemosensory stimuli. We then used computational methods to identify various stimuli by analyzing neural activity. Specifically, we used a combination of population-level activity statistics (e.g., average, standard deviation, frequency-based measures) and graph-theoretic features of functional network structure (e.g., transitivity, which is the existence of strongly connected triplets of neurons) to accurately predict salt stimulus. Our method is general and can be used across species, particularly in instances where the identities of individual neurons are unknown. These results also suggest that neural activity downstream of the sensory periphery contains a signature of changes in the environment.​

## Introduction

Animals have evolved mechanisms for encoding a multitude of chemical stimuli encountered in the environment. The neural circuitry encoding odor and taste information in vertebrates and invertebrates is thought to include both labeled lines and combinatorial activity patterns. Odor information is initially filtered by olfactory sensory neurons that are organized into specific expression zones within the vertebrate olfactory epithelium [1], or into specific invertebrate sensilla that are selective for pheromones [2], food odors [3], acids [4], oviposition cues [5], or toxic odors [6]. In both cases, olfactory information is then relayed to specific glomeruli and higher-order centers in the brain [7,8]. Similarly, taste information in both mice and flies is represented by spatial patterns of neural activity, likely using combinatorial coding [9–11]. While these studies highlight how chemical information is encoded in the periphery and early cortical areas, its processing and representation within higher brain centers remain poorly understood. One way of addressing this problem would be to monitor neural activity of the entire circuit in an intact nervous system as the animal experiences changes in its chemical environment. Neural features that encode these changes could then be extracted.

The *Caenorhabditis elegans* (*C*. *elegans*) nervous system consists of just 302 neurons connected by identified chemical and electrical synapses [12,13], and is therefore ideally suited for recording neural activity across a large part of the entire network. *C*. *elegans* neurons express rapidly activating voltage-gated calcium channels, and thus changes in neuronal calcium approximately correlate with neuronal depolarization [14–16]. We previously monitored neural activity in a small population of neurons using genetically encoded calcium indicators and showed that *C*. *elegans* sensory neurons encode the identity and concentration of chemical stimuli using a combinatorial code [17,18]. Moreover, large-scale activity measurements of the *C*. *elegans* nervous system are enabled by two key innovations: 1) custom-designed microfluidic devices that can immobilize adult animals and precisely deliver chemical stimuli while neural activity is being recorded [19,20], and 2) genetically encoded calcium indicators that localize to the nucleus; this restricted fluorescent signal makes it easy to distinguish between adjacent neuronal nuclei [21].

Previous analyses of *C*. *elegans* whole-brain imaging data used principal components analysis (PCA) to show that neural activity lies in a “low-dimensional manifold” [22–24]. For example, Kato et al. [22] showed that the *C*. *elegans* neural network likely exists in a few global states that represent locomotory commands, such as forward movement, reversals, turns, and others. Nichols et al. [23] and Skora et al. [25] revealed low-dimensional neural activity patterns associated with specific physiological and behavioral states, such as sleep and starvation. However, Scholz et al. [26] found evidence that neural dynamics may have higher dimensionality than previously thought, suggesting that many more neurons are engaged to drive behavior. If this is the case, approaches to understand the brain must consider the entire neural network to provide a comprehensive analysis.

Therefore, here we ask: can we predict stimulus properties using graph-theoretic features of neural interactions recorded during stimulus onset or offset? Graphs are typically used to capture pairwise interactions between nodes (e.g., neurons) connected by edges (e.g., functional interactions). Graphs have also been used to uncover structure-function relationships in physical, biological, social, and information systems [27–29]. By viewing the nervous system as a graph, we hypothesize that we will uncover complex patterns of activity that are generally missed when neurons are treated as independent units. To test this approach, we monitored the responses of at least 50 head neurons while subjecting adult *C*. *elegans* to one of five chemical stimuli at one of two concentrations. In *C*. *elegans*, head neurons include olfactory and gustatory sensory neurons, several downstream interneurons and command interneurons that direct locomotion, and some motor neurons [12]. Next, we computed how two stimulus properties–a chemical’s valence (i.e., attractant or repellent) and identity (i.e., chemical structure)–affected neural activity across the network. We observed that both graph-theoretic and activity-based features successfully discriminated between various stimuli. We validated these results using two independently collected data sets and machine learning classification. However, neither graph-theoretic features nor activity statistics were modulated by stimulus valence. We also found that chemical identity weakly altered the subnetworks composed of putatively excitatory or inhibitory interactions in isolation, suggesting that network-wide interactions between inhibition and excitation more strongly define the representation of a chemical in the nematode brain. Further, when we decoupled correlative from causative interactions amongst putative sensory neurons, graph-theoretic features almost doubled the accuracy of predicting stimulus identity compared to using activity features alone, thereby improving our method’s robustness and suggesting that chemical identity strongly modulates functional interactions in the *C*. *elegans* nervous system.

## Results

**Fig 1:**
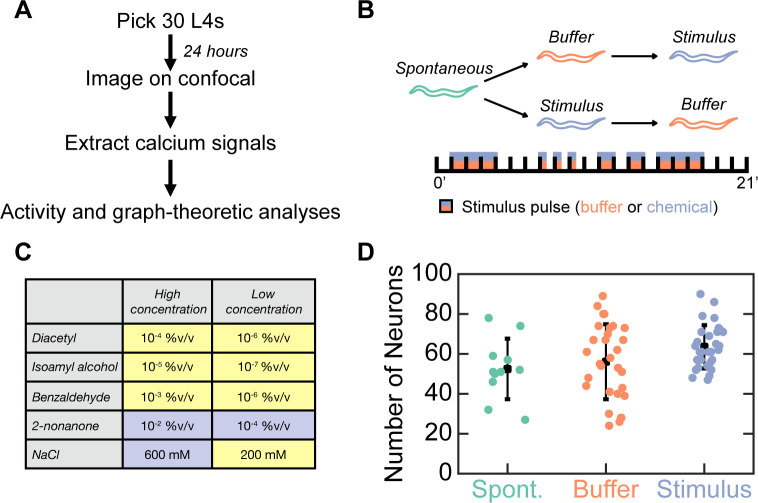
Whole-brain imaging experiments and analysis. A) Schematic showing the experimental protocol. Thirty stage L4 animals were picked onto a plate covered with OP50 24 hours before each experiment. Animals, as 1-day old young adults, were stimulated in an olfactory chip and their neural responses were imaged on a Zeiss Airyscan 880. Fluorescence traces were extracted from each video, and subsequently analyzed. B) Animals were imaged in three 21 minute-long sessions: Spontaneous, Buffer, and Stimulus. Buffer and Stimulus sessions had seven pulses of M9 buffer or one of 10 chemicals, respectively (indicated by the dual-color shading). Animals only experienced M9 buffer or a single chemical stimulus on any given 21-minute long session using an identical stimulus protocol (see dual-color boxes). C) Table showing the chemicals (attractants are colored yellow, repellents are violet) and concentrations tested. Three animals were tested per stimulus condition. %v/v refers to % vol/vol. D) We recorded a similar number of neurons during Spontaneous (green), Buffer (orange), and Stimulus (blue) sessions (p = 0.0947 by Kruskal-Wallis test). Each color dot is the number of neurons recorded in a single worm, and the black squares and lines indicate the mean and standard deviation for a given session type. N = 12, 30, and 30 animals for Spontaneous, Buffer, and Stimulus sessions, respectively.

We performed whole-brain calcium imaging in 30 worms immobilized in an olfactory chip–a microfluidic device that permits near-instantaneous switching between two fluid flows (Fig 1A; [19]). Each worm experienced three 21-minute long imaging sessions: one without stimulation (“Spontaneous”, where M9 buffer was present but not switched), one with buffer changes around the animal’s nose (“Buffer”, where we switched between two streams of M9 buffer), and one with chemical stimulation (“Stimulus”, where we switched between M9 buffer and an odorant or tastant that was diluted in M9 buffer). Both Buffer and Stimulus sessions consisted of seven pulses that lasted 30 seconds, 1 minute, or 3 minutes (Fig 1B; modified from [30]). We exposed worms to one of 10 conditions: high or low concentrations of one of five chemical stimuli (Fig 1C; S1–S5 Videos). Stimuli were either innately attractive or repellent, as determined by previous chemotaxis and drop assays (Fig 1C; [31,32]). We monitored and tracked the activity of each neuron individually within a session, but we did not identify the same neurons across animals or sessions. A mean of 52, 56, and 64 neurons were active during Spontaneous, Buffer, and Stimulus sessions, respectively, indicating that our imaging setup was able to record from similar numbers of neurons during each session (Fig 1D). All Stimulus sessions activated similar numbers of neurons, regardless of stimulus identity (S1 Fig). Some neural responses were locked to the onset or offset of the stimulus (i.e., a stimulus switch) in the Buffer and Stimulus sessions, and were likely sensory or interneurons involved in the detection and behavioral response to chemical, mechanosensory, or both forms of stimulation [20]. Other neurons may be interneurons or motor neurons involved in motor commands [22].

Previous studies have shown that spontaneous activity in the nervous system of an immobilized worm lies in a “low-dimensional” PCA space [22,23,25]. Indeed, our analyses also suggest that during the Spontaneous sessions, neural activity loops through a low-dimensional manifold, where the first three principal components (PCs) capture a mean of 56% of the variance in our 21-minute imaging sessions (S6A, S6D and S6E Fig). However, we found that population activity during any type of stimulation–either Buffer or Stimulus changes–exhibited higher-dimensional network dynamics than observed in an unstimulated worm (S6B–S6E Fig). Specifically, the first three PCs captured a mean of only 51% and 49% of the variance in Buffer and Stimulus sessions (S6D and S6E Fig) and, instead of smooth trajectories through PCA space, we observed jumps between different regions of state space (S6B and S6C Fig). This agrees with Scholz et al. [26], who found that a non-stimulated, but moving, worm exhibits network dynamics that are not well-explained by the first three PCs. We also find that the participation ratio–an alternative measure of dimensionality which describes how many dimensions capture ~86% of the total variance [33]–is larger in Stimulus sessions compared to Buffer and Spontaneous sessions (S6F and S6G Fig). This suggests that, perhaps unsurprisingly, the dynamics of a population of neurons is more complex during stimulus processing, and we propose to dissect its dynamics by focusing on two fundamental components of neural network dynamics: the activity of individual neurons, and the interactions between them.

### Neural activity statistics are not significantly modulated by stimulus valence nor identity

We first tested if traditional measures of neural activity exhibited predictable changes with respect to different stimulus properties. We considered two ecologically relevant stimulus properties: 1) Identity (benzaldehyde, diacetyl, isoamyl alcohol, 2-nonanone, or NaCl), and 2) Valence (attractive or repellent). We focused on statistical measures (e.g., average and standard deviation of normalized population-level neural activity) and other measures that capture temporal dynamics (Fourier-based analysis of frequency spectra [34]). Each cell’s activity was normalized to the peak value it attained during the 21-minute session, and all measures were normalized to their pre-switch (i.e., before stimulus onset or offset) values.

We assessed the robustness of our results in two ways. First, to correct for multiple comparisons we computed the effective number of variables (m_eff_) that would correct an alpha value of 0.05 (corrected alpha = 0.05/m_eff_)[35]. This approach was used because many features are correlated–thereby violating the assumptions of a standard Bonferroni correction. Second, to test the reproducibility of our data and the generality of our results we performed experiments on a second set of 24 worms (Data Set 2). Data for this second set was gathered similarly to Data Set 1 (i.e., the first 30 worms we studied), except we did not expose any worms to diacetyl. In other words, Data Set 2 encompasses whole-brain calcium imaging videos of worms exposed to benzaldehyde, isoamyl alcohol, 2-nonanone, or NaCl. We report as statistically significant only those features that survived the m_eff_-based Bonferroni correction in both Data Sets 1 and 2.

**Fig 2:**
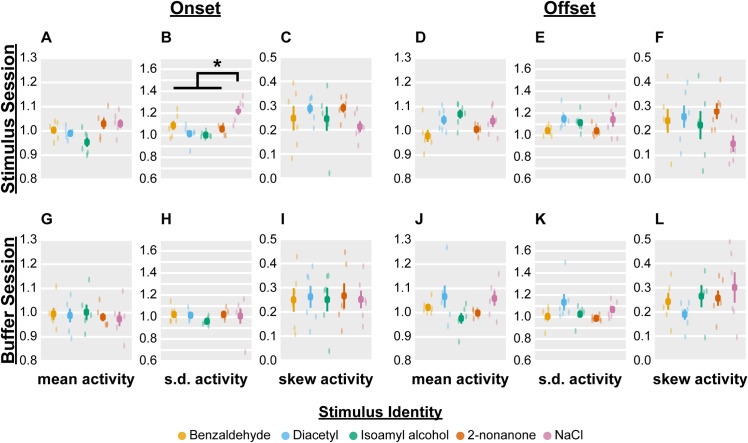
Neural network activity features are not modulated by stimulus identity. Activity features were computed upon stimulus onset (A, B, C, G, H, I) or offset (D, E, F, J, K, L) of an odorant/tastant (A, B, C, D, E, F) or M9 buffer stimulus (G, H, I, J, K, L). “Mean activity” refers to the mean change of mean population activity relative to baseline, “s.d. activity” refers to the mean change of the standard deviation of population activity relative to baseline, and “skew activity” refers to the mean change of the skewness of population activity relative to baseline, where baseline is the 30-second period preceding chemical onset or offset. Each color dot is the mean value across all seven pulses for a single worm, and the dark squares and lines indicate the mean and standard error of the mean across all worms (n = 6 worms per stimulus). p > 0.05 for all comparisons after Bonferroni correction with effective number of variables meff = 27.1, except panel B (p = 1.1e-4). However, note that p = 0.02 for panel B in Data Set 2, which does not survive Bonferroni correction with meff = 6.2 (hence, no red star). We used the likelihood ratio test on full and null generalized linear-mixed effects models (GLMEs), where the former included stimulus identity as a fixed effect, to compute the aforementioned p-values. We then used pairwise F-tests for Data Set 1 on the stimulus coefficients of the full GLME to compute pairwise significance, which is indicated by * and the bar connecting NaCl and the rest of chemicals in panel B. The F-tests used Bonferonni correction based on the total number of distinct pairwise comparisons (i.e., alpha = 0.05/10).

With this more stringent definition of statistical significance, we found that broad neural activity features did not distinguish between stimulus identity nor valence at stimulus onset (Figs 2A–2C, S2 and S4) or offset (Figs 2D–2F, S3 and S5). This was true of both Data Sets 1 and 2. For example, the tastant NaCl induced more variable neural activity than the odorants on stimulus onset in Data Set 1 (Fig 2B–black asterisk indicates significant modulation in Data Set 1, S2 Fig), but not on Data Set 2 (Fig 2B–lack of red asterisk, which indicates no modulation in Data Set 2, S4 Fig), suggesting that this was not a robust finding. Additionally, activity features of either stimulus onset or offset were not selectively altered by attractant or repellent stimuli (S2–S5 Figs). Of all the activity features we analyzed, we found that skewness always dropped to ~0.3 in Stimulus and Buffer sessions, though not in a stimulus- or valence-dependent manner (S2–S5 Figs). This decrease in relative skewness (i.e., skewness post-stimulus / skewness pre-stimulus) suggests that each neuron’s distribution of activity was, on average, skewed prior to stimulus addition or removal, and became more symmetric (i.e., less skewed) upon stimulus addition or removal. It is an open theoretical question of “why” skewness may drop after stimulus, but in any case, we note that this decrease is agnostic to stimulus identity. Finally, there was no significant difference for any activity feature in Buffer trials (Figs 2G–2L, S2–S5), which indicates that these measures are also insensitive to changes in buffer flow around the worm’s nose. Thus, broad, population-level activity features are not distinctly modulated by stimulus properties.

### Computing graph-theoretic features of neural activity

We next asked if different stimulus properties could be better distinguished based on interaction patterns between neurons, instead of activity statistics of neuronal populations. Graph-theoretic features capture complex interactions amongst groups of neurons. For example, some features quantify how well a network can be divided into relatively independent clusters, such that neurons in one cluster mostly interact with other neurons within the same cluster, and sparsely interact with neurons in all other clusters (this feature is called modularity [36]). Other features capture how quickly information can spread through the network, such that signals in one part of the network can be received and processed by another part of the network (one such feature is the largest eigenvalue of the adjacency matrix) [37,38]. Graph-theoretic features have been previously used to characterize activity changes in coarse brain networks (e.g., where nodes represent entire brain regions or large populations of neurons; reviewed by [37,39]), and have recently been used to analyze whole-brain activity at the single neuron level [40–42].

**Fig 3:**
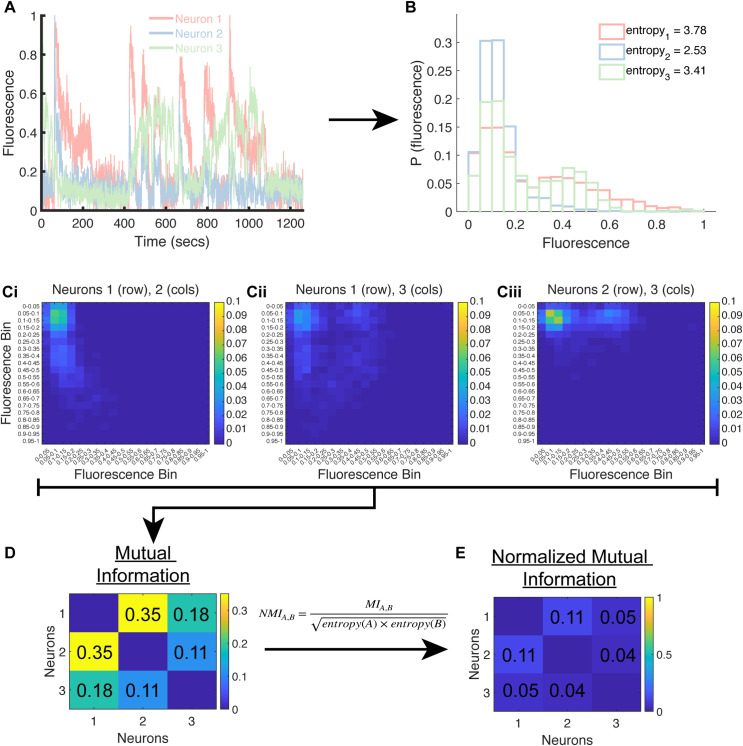
Example of generating an adjacency matrix from the time-series activity of three neurons. A) Activity of three neurons from one worm experiencing 10^−2^ 2-nonanone. The three traces are normalized to their max (i.e., each trace is divided by the maximum fluorescence value it achieved in the 21-minute long imaging session). B) Time series are binned into histograms using bins of size 0.05. The legend shows the entropies of the histograms of each neuron from panel A. C) The joint probability distributions of the histograms in panel B for: Ci) Neuron 1 (rows) and Neuron 2 (columns), Cii) Neuron 1 (rows) and Neuron 3 (columns), and Ciii) Neuron 2 (rows) and Neuron 3 (columns). D) The mutual information (MI) between each pair of histograms is computed and then normalized to a maximum of 1 via the equation shown above the arrow, to yield the normalized mutual information (NMI) between each pair of neurons. E) The matrix shown in E is the adjacency matrix used in further graph-theoretic analyses. In this example, we used the entire time series to compute an adjacency matrix, but in our analyses we used 30-second long periods of time.

To characterize interactions between neurons, we first need to infer interactions between neurons based on their individual time series traces. For example, if neuron B’s activity rises shortly after neuron A’s activity rises, then we may infer a functional interaction between neurons A and B. Formally, to predict interactions, we computed the normalized mutual information (NMI; [43]) between the activity vectors of every pair of neurons in a 30-second period around a stimulus switch (i.e., stimulus onset or offset; see Fig 3 for an example application of this procedure). NMI measures how much information one variable contains about another variable, which in this context reveals a putative interaction between two neurons on the basis of their activity vectors (see Materials and Methods, Fig 3; S7 Fig for examples). We computed the NMI between all pairs of *n* neurons in a given worm during a 30-second period of interest (either before or after a stimulus switch). We focused on a 30-second period for two reasons: 1) an animal can begin to move toward or away from an attractant or repellent, respectively, well within 30 seconds [30,44], and 2) sensory neurons that detect chemical stimuli tend to reach their maximum response within ~10 seconds of stimulus onset [20]. Thus, analyzing 30 seconds following a stimulus switch should be sufficient to capture the representation of ecologically relevant information in neural activity [20,32,44].

We then computed a weighted, undirected graph *G = (V*,*E)*, where *V* is the set of nodes (unlabeled neurons) and *E* is the set of weighted edges (inferred functional interactions) between neurons. Each edge weight (between 0 and 1) equals the NMI between the two neurons, where a larger number implies a stronger interaction (Fig 3D). Pearson’s correlation (PC) has been used [45] to generate functional connectivity networks with both positive and negative weights (as well as 0 for uncorrelated neurons, which is rare in practice); positive weights suggest that both neurons increase or decrease their activity together (e.g., one excites the other), and negative weights imply that as one neuron increases its activity, the other neuron’s activity decreases (e.g., one inhibits the other). As a group, GCaMPs are known to faithfully reflect increases in a cell’s internal calcium concentration, and thus its excitation. However, GCaMPs were not optimized to reflect a decrease in calcium concentration or neuronal hyperpolarization (e.g., see [46,47]). Further, many graph-theoretic analyses require that all edge weights be non-negative, and we found that 47.8% of edges in Data Set 1 and 47.5% of edges in Data Set 2 had a negative weight when we used Pearson’s correlation. Thus, we did not use weights from Pearson’s correlation, though we did use it to study the putatively excitatory and inhibitory subnetworks independently.

From each graph we extracted network features that capture different interaction patterns at both the local and global network scales. We focused on five classes of network measures, namely basic structure, functional segregation, functional integration, centrality, and resilience (summarized in S1 Table and [37]). Basic structure refers to general aspects of the graph, such as the largest eigenvalue of the adjacency matrix or the median weight of the network, which indicates how strongly nodes interact with one another. Functional segregation measures the extent of interconnection amongst small groups of nodes, or modules, and encompasses the network’s transitivity and modularity–in a more modular network, nodes cluster into groups that are connected strongly. Functional integration indicates how strongly interconnected the network is; a representative measure is the average shortest path distance between pairs of nodes within the network, which indicates how short, on average, is the path connecting one node to any other node. Centrality measures how interconnected a node is to other parts of the network and can be assessed, for example, using the average betweenness centrality–the average fraction of shortest paths linking any two nodes that pass through a given node. Finally, measures of resilience to perturbations, such as lesions, includes the average assortativity coefficient–the average correlation coefficient between the degrees of any two connected nodes [37].

To highlight changes in a graph-theoretic property after a stimulus switch and account for any dependence on network size [48], we used the same normalization scheme we used for neural activity features (i.e., we divided the post-switch value of the feature by its pre-switch value). This was a critical normalization scheme, as the worm’s neural network was dynamic, even in the absence of stimulation (S8 Fig). Thus, we report how the addition or removal of a stimulus affected ongoing patterns of neural network activity.

### Graph-theoretic features can better distinguish stimulus identity

For each stimulus property we tested the extent to which any graph-theoretic feature significantly changed in response to a stimulus switch. Unlike activity features, network features were significantly modulated by stimulus identity, though only on stimulus onset.

**Fig 4:**
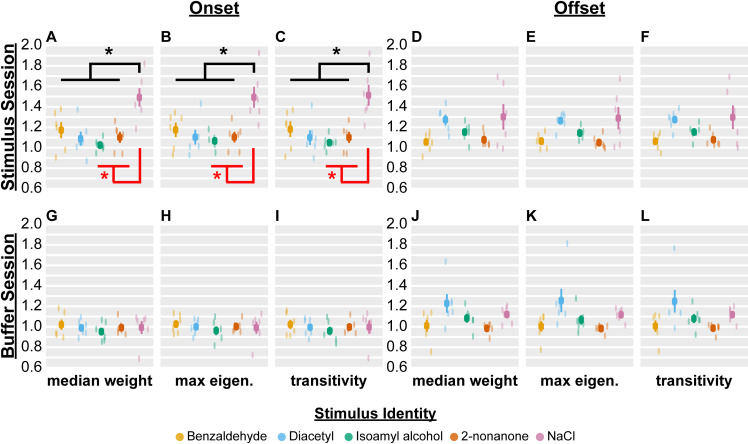
Neural network graph features differ between NaCl and the odorants isoamyl alcohol and 2-nonanone on stimulus onset. “Median weight” refers to the change in median entry of adjacency matrix, “max eigen.” refers to the change in its max eigenvalue, and “transitivity” to the change in its weighted number of triangles (i.e., triplets of neurons interacting with each other) relative to baseline. All three features are modulated by stimulus identity on stimulus onset (A, B, C), but not offset (D, E, F); they are also not modulated by the addition (G, H, I) or removal (J, K, L) of M9 buffer. Each color dot is the mean value across all seven pulses for a single worm, and the dark squares and lines indicate the mean and standard error of the mean across all worms (n = 6 worms per stimulus). p > 0.05 for all comparisons after Bonferroni correction with effective number of variables meff = 27.1, except panels A—C (black *, p = 2.5e-4, 7.4e-4, and 3.9e-4). Furthermore, p < 0.05 after Bonferroni correction with meff = 6.2 for panels A—C in Data Set 2 (red *, p = 5.4e-3, 7.7e-3, and 6.9e-3). We used the likelihood ratio test on full and null generalized linear-mixed effects models (GLMEs), where the former included stimulus identity as a fixed effect, to compute the aforementioned p-values. We then used pairwise F-tests for both Data Set 1 and 2 on the stimulus coefficients of the full GLME to compute pairwise significance at p < 0.05, which is indicated by black * for Data Set 1, * for Data Set 2, and the bar connecting NaCl and the rest of chemicals in panels A—C. The F-tests used Bonferonni correction based on the total number of distinct pairwise comparisons (i.e., alpha = 0.05/10 for Data Set 1, and 0.05/3 for Data Set 2). Together, the asterisks indicate that the NaCl-induced network differs from the network induced by isoamyl alcohol and 2-nonanone in both data sets. Though NaCl also induced larger feature values than diacetyl in Data Set 1 (black * in A—C), this could not be tested in Data Set 2 (lack of red * in A—C), which lacks diacetyl sessions.

There were six graph-theoretic features that reliably changed on stimulus onset in both data sets, namely the average network weight, median network weight, max eigenvalue, average clustering coefficient, transitivity, and average local efficiency (Figs 4A–4C, S9 and S11, and S2 Table). The neural network of worms exposed to NaCl had the largest value for all six features, although significantly larger values were seen only in comparisons to the neural network of worms exposed to isoamyl alcohol and 2-nonanone (Fig 4A–4C, black and red asterisks, which suggests significant modulation in Data Sets 1 and 2). This indicates that the NaCl-induced network could efficiently transmit information across the nervous system by strengthening neural interactions (i.e., increasing average and median network weight). Furthermore, this network had the largest transitivity (i.e., average strength of interactions between connected triplets of neurons) and the largest max eigenvalue (the larger the max eigenvalue, the more easily signal spreads through a network), suggesting a unique combination of strong local connectivity and efficient global reach, which is often observed in small-world networks [49]. These results also suggest that the neural networks of worms exposed to benzaldehyde had intermediate network weights and max eigenvalues compared to those observed with NaCl, isoamyl alcohol, and 2-nonanone. No graph-theoretic features were modulated by buffer onset or offset (Figs 4, S9–S12 and S2 Table).

These results were only significant when we used a time window of 30 seconds around stimulus onset, but not when we used time windows of 15 or 23 seconds (S17 and S18 Tables). Furthermore, if we used a time window of 30 seconds, these results were significant when we used an NMI bin (i.e., bin size used to make histograms) of 0.05 or 0.1, but not 0.2 (S19 and S20 Tables). Our observations that 23 or 15 second-long windows fail to reveal substantial changes in the graph-theoretic results could have two interpretations. Biologically, it could mean that substantial network reconfigurations occur somewhere between 23–30 seconds of stimulus onset, in part due to the response of late-acting sensory and interneurons which are known to be active for tens of seconds after stimulus presentation or removal [20,34,50]. On the other hand, it could also suggest that we are not able to discriminate among the distribution of states of each neuron (which is required to calculate NMI) at our current imaging rate unless we use a window size of about 30 seconds. One alternative to test the latter hypothesis would be to use a higher imaging rate, thereby acquiring a similar number of data points within a shorter period. This suggests that it is important to capture high-resolution neural activity over tens of seconds to accurately reconstruct the network of functional interactions induced by chemical stimuli.

**Fig 5:**
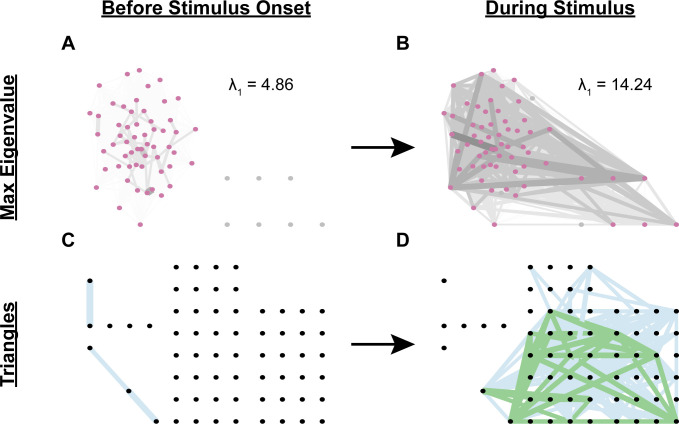
NaCl induces an increase in max eigenvalue and number of (strong) triangles. The networks depicted here, for one example worm, show neurons (circles) connected by lines (edges). In Panels A and B, the normalized mutual information (NMI) between any two neurons is indicated by both edge shading and thickness, where darker and thicker lines indicate larger NMI. Panels A and B show the network’s max eigenvalue (λ1), and Panels C and D depict the strongest (i.e., NMI > = 0.4) interactions in the network (hence, why many edges are missing), with those in green indicating the presence of a triangle (i.e., a triplet of connected neurons whose total weight > = 1.8) and those in blue its absence. Before the worm is exposed to 200mM NaCl, the neural network is composed of weak interactions, producing a low max eigenvalue (A), and no strong triangles (C). Once exposed to 200mM NaCl, the worm’s neural network has many stronger interactions, increasing its max eigenvalue (B), and forming several strong triangles (D). Edges in panels A and B were assigned to 0.2-wide bins from 0 to 1. All edges less than 0.4 were removed from panels C and D for the purpose of visualization. Neuron positions were fixed between panels A and B, and C and D, such that A can be directly compared to B, and C can be directly compared to D.

Graph-theoretic features quantitatively captured differences in network structures that emerged during stimulus presentation. To illustrate these differences more intuitively, we visualized how the networks changed in response to stimulus onset. Fig 5 shows the network of one worm before the onset of 200 mM NaCl (Fig 5A and 5C), and during the first 30 seconds of the first pulse of NaCl (Fig 5B and 5D). In the latter, there is a general increase in edge weight (i.e., strength of interaction between neurons, depicted by darker and thicker lines connecting neurons), more neurons are connected with the rest of the network (thereby increasing the max eigenvalue), and triplets of neurons are more likely to be strongly connected (i.e., a larger transitivity, depicted with green lines between strongly connected triplets). Due to the normalization scheme employed, changes in the values of graph-theoretic features may appear to be mild; however, as illustrated, these changes capture significant differences in connectivity patterns between neurons and may be biologically informative.

### Using machine learning methods to predict stimulus identity

We used machine learning to test how well neural activity features (Fig 2) and graph-theoretic features (Fig 4) could predict stimulus identity on the first stimulus pulse, when the animal has not undergone any adaptation. We also combined graph-theoretic and neural activity features to see if they provided distinct information that could collectively improve classification accuracy. We did not consider stimulus valence, as it did not modulate activity or network properties. We used a logistic regression classifier, which is a simple and commonly used classifier with no built-in assumptions about the distribution of the data. The training and testing sets were constructed using nested leave-one-out cross-validation to tune the regularization hyperparameter *C*. This was followed by a permutation test to assess the classifier’s performance against an empirically derived chance level of accuracy (see Materials and Methods). We attempted classification using scaled or unscaled features, where the former had zero mean and unit variance.

**Fig 6:**
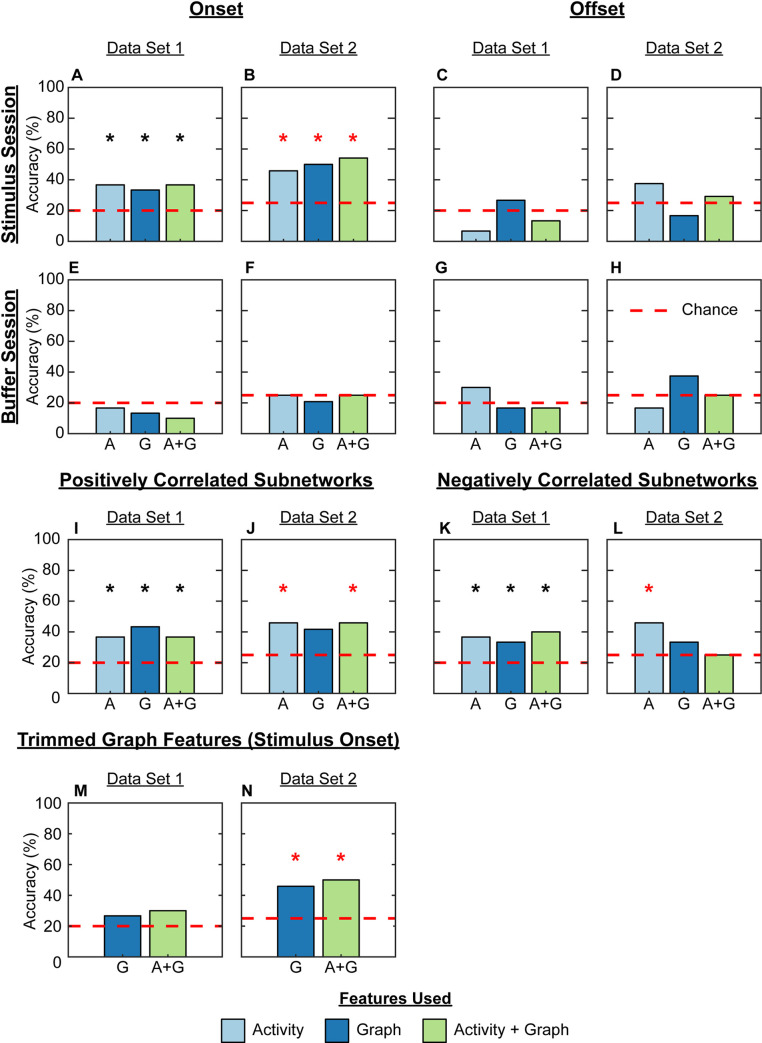
Activity and graph features can distinguish between chemical stimuli. A logistic regression classifier was trained to identify stimulus identity using leave-one-out cross-validation on Data Set 1 (A, C, E, G, I, K, M) and 2 (B, D, F, H, J, L, N) for stimulus onset (A, B, E, F, I, J, K, L, M, N) and offset (C, D, G, H). We used Activity features (light blue bars), Graph features (dark blue bars), and both Activity and Graph features together (green bars). The classifier achieved statistically significant above-chance accuracy on stimulus onset for both data sets with any combination of features (A, p = 3.0e-2, 2.0e-2, and 2.0e-2, for A, G, and A+G, respectively; B, p = 3.0e-2, 1.0e-2, and 1.0e-2 for A, G, and A+G, respectively). Activity features, and the combination of Activity and Graph features, were most predictive when computed during stimulus onset on the positively correlated subnetwork of putatively excitatory interactions (I, J; p = 3.0e-2, 1.0e-2, 3.0e-2 for A, G, and A+G in panel I; p = 3.0e-2 and 3.0e-2 for A and A+G in panel J). For the negatively correlated subnetwork of putatively inhibitory interactions, only the Activity features were useful for both data sets (K, L; p = 3.0e-2, 3.0e-2, and 1.0e-2 for A, G, and A+G in panel K, and p = 3.0e-2 for A in panel L). (M, N) We removed the six statistically-significant Graph features shared by Data Sets 1 and 2 to test whether these features were important for classification; the ensuing set of features are called trimmed Graph features. Classification was at chance accuracy when using the trimmed Graph features alone or in combination with Activity features for Data Set 1 (M; no features were standardized), and above-chance accuracy when using trimmed Graph features alone or in combination with Activity features for Data Set 2 (N; p = 1.0e-2 for both asterisks; trimmed Graph features were not standardized, but the combination of trimmed Graph and Activity features were standardized). The decision to standardize features in panels M and N was based on which produced the highest accuracy in panels A and B. The dashed red line refers to chance accuracy, which is 20% for Data Set 1 (5 chemical stimuli) and 25% for Data Set 2 (4 chemical stimuli). Statistical significance was assessed using a permutation test with n = 100 permutations. N = 30 worms for Data Set 1, 24 worms for Data Set 2. Black * and red * refer to classifiers with accuracies significantly above chance according to permutation test in Data Sets 1 and 2, respectively.

The logistic regression classifier performed well on predicting stimulus identity on onset, but not offset (Fig 6A–6D and S9 Table), and both activity and graph-theoretic features performed significantly better than chance on both data sets. In general, accuracy was higher on Data Set 2 than 1, partly because the former only had to classify among fewer classes (Data Set 1 has 5, while Data Set 2 has 4 classes) (Figs 6A, 6B, and S13). In particular, accuracy was moderate on Data Set 1 when using graph-theoretic features (33% accuracy, chance: 20%, permutation accuracy: 14±8%, p-value = 0.02, Fig 6A) or activity features alone (37% accuracy, chance: 20%, permutation accuracy: 17±10%, p-value = 0.03, Fig 6A). Adding neural activity features to graph-theoretic ones did not change the accuracy by much (37% accuracy, permutation accuracy: 14±7%, p-value = 0.02, Fig 6A). We found that classification on Data Set 2 was high when using graph features (50% accuracy, chance: 25%, permutation accuracy: 17±11%, p-value = 0.01, Fig 6B) or activity features alone (46% accuracy, permutation accuracy: 19±10%, p-value = 0.03, Fig 6B and S9 Table), and improved by combining both sets of features (54% accuracy, permutation accuracy: 18±11%, p-value = 0.01, Fig 6B). Finally, we did not exceed chance accuracy when classifying stimulus identity from features computed on the buffer sessions (Fig 6E–6H and S9 Table).

These results were sensitive to the time window and the NMI bin size we used. Our classifier’s accuracy was highest (33% for Data Set 1, 50% for Data Set 2) when we considered periods of 30 seconds around stimulus onset and used fine neural activity bins of size 0.05 to infer neural interactions (S21 and S22 Tables). Changing the time window or NMI bin changed accuracy on Data Set 1 to a low of 17% and high of 33% (S21 Table), and on Data Set 2 to a low of 13% and high of 50% (S22 Table). This is consistent with our sensitivity analysis for the statistical significance of graph-theoretic features (S19 and S20 Tables), which showed that high-resolution neural activity over longer periods of time are needed to reconstruct the network of interactions induced by chemical stimuli.

We next sought to determine whether the six graph-theoretic features that were significantly modulated by stimulus identity on both Data Sets 1 and 2 were used by our classifier to discriminate stimulus identity; if so, this would reiterate that different chemical stimuli evoke different changes in the *C*. *elegans* neural network. We reasoned that if these features were indeed useful for classification, then by *not* using these six features, our classifier would not be able to achieve above-chance accuracy. Hence, we performed classification using non-statistically-significant Graph features on Data Set 1 (Fig 6M), non-statistically-significant Graph features and all Activity features on Data Set 1 (Fig 6M), non-statistically-significant Graph features on Data Set 2 (Fig 6N), and non-statistically-significant Graph features and all Activity features on Data Set 2 (Fig 6N). The results show that dropping the statistically-significant Graph features reduces classification accuracy to chance level on Data Set 1, with or without including Activity features (Fig 6M). Moreover, the non-statistically-significant Graph features achieve above-chance accuracy on DS2 (46%; Fig 6N), though this is a slight drop in accuracy compared to when using the statistically significantly modulated Graph features (50%; Fig 6B). Similarly, the non-statistically-significant Graph features with the Activity features together achieved above chance accuracy on DS2 (50%; Fig 6N), though this is also a slight drop in accuracy compared to when the statistically significantly modulated Graph features were used with the Activity features (54%; Fig 6B). Hence, the statistically significantly modulated Graph features seem to be used by the classifier to improve discrimination of stimulus identity, as their inclusion consistently produced above-chance classification accuracy on both Data Sets 1 and 2 (Fig 6A and 6B), even if they do not receive the largest weights in the logistic regression classifier (S23–S28 Tables).

Thus, both activity and graph-theoretic features alone appear capable of quantitatively discriminating identity on stimulus onset, with some gain when considering them together.

### Contributions of putative excitatory and inhibitory subnetworks to the discrimination of stimulus identity

NMI can detect non-linear interactions between two signals but is unable to determine the sign of the interaction. Thus, two neurons with high NMI may be positively or negatively correlated. The sign of the correlation is important because it implies different types of interactions, both of which are biologically meaningful–positive correlations are thought to indicate excitation, whereas negative correlations may imply inhibition.

We next sought to determine whether positively or negatively correlated subnetworks contributed more towards discriminating stimulus identity. We computed adjacency matrices based on Pearson’s correlation (PC) and used the sign of those networks to split the NMI-based networks into two subnetworks: one composed of putatively excitatory interactions, and the other composed of putatively inhibitory interactions. This enabled us to use PC to detect each subnetwork and to then use NMI to capture non-linear neuronal interactions within each subnetwork. We found that a few network features changed for both subnetworks in an identity-dependent manner, and that none of these features were shared between the excitatory and inhibitory subnetworks (S8 Table). The positively correlated subnetwork’s clustering coefficient and local efficiency were significantly modulated on stimulus onset in Data Sets 1 and 2, just like the full network. On the other hand, the negatively correlated subnetwork’s modularity was significantly affected by stimulus identity on stimulus onset in each Data Set. Moreover, just as in the full network, there were no identity-dependent changes on stimulus offset, buffer onset, or buffer offset (S7 and S8 Tables). This suggests that the positively and negatively correlated subnetworks each partly drove the organization of the full network, though neither alone was responsible for the most prominent changes detected in the full network (Fig 4).

We then tried to classify stimulus identity using network features solely derived from one subnetwork at a time. We found that, unlike the full network, which could use graph features alone to classify stimulus identity for both data sets, graph features based on either the positively or negatively correlated subnetworks alone could not achieve a level of accuracy that was significantly greater than chance on Data Set 2 (42% and 33% accuracy for the positively and negatively correlated subnetworks, chance: 20% and 25%, permutation mean accuracy ± standard deviation: 19±11% and 21±11%, p-values = 0.06 and 0.18; Fig 6J and 6L, and S13 and S14 Tables). This suggests that stimulus identity most strongly modulates the interplay between excitation and inhibition in the *C*. *elegans* neural network, and not just one subnetwork in isolation.

Overall, these additional analyses demonstrate that a graph-theoretic analysis can be used to understand how excitation and inhibition interact to coordinate a neural network’s response to different stimuli. We speculate that other parameters introduced when extracting features (e.g., inferring directionality) may provide further biological insight.

### Trivially high NMI between putative sensory neurons impairs classifier accuracy and generalization

An important caveat to the analyses presented thus far is that neural interactions are inferred based on correlation, as opposed to causation. This can be particularly problematic when there are unobserved variables (e.g., neurons, environmental signals, brain states) that drive functional interactions between observed neurons in our network. For instance, if unobserved neuron A excites observed neurons B and C, then neurons B and C will have large NMI in the network, even though B and C may not directly interact. While there are computational approaches that attempt to disentangle these two effects (see Discussion), here we sought to study the effect of removing correlative effects driven by the environmental signal itself. Specifically, the addition or removal of a stimulus will induce some sensory neurons to become simultaneously excited or inhibited, which will lead to a large NMI between these sensory neurons, even though they likely do not directly interact. We hypothesized that removal of these trivially strong interactions between putative sensory neurons could improve our ability to detect robust, stimulus-dependent patterns of network organization.

**Fig 7:**
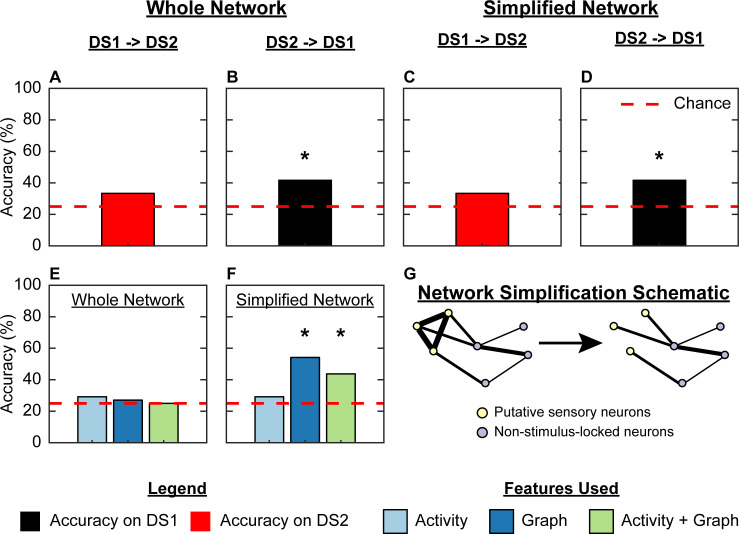
The Simplified Network made by removing trivial interactions between putative sensory neurons improves robustness of the logistic regression classifier, and shows that Graph features alone are capable of discriminating stimulus identity. Graph features from the Whole Network allowed above-chance accuracy when training on Data Set 2 (DS2) and testing on Data Set 1 (DS1; B, * p-value = 0.04), but not vice-versa (A); the same was true of Graph features from the Simplified Net- work, which produced the same accuracies as the Whole Network (C,D, * p-value = 0.04). Moreover, Graph features from the Simplified Network led to above-chance classification accuracy on the combined data set of 48 worms (E,F, * p-value = 0.01), while Activity features could not be used to exceed chance-accuracy. The process of network simplification is depicted in panel G via a cartoon, where we removed the (trivially) strong edges between neurons correlated to stimulus onset (i.e., “putative sensory neurons”, yellow circles). No features were standardized. The dashed red line refers to chance accuracy of 25%. Statistical significance was assessed using a permutation test with n = 100 permutations. N = 24 worms for Data Set 1 (all worms except those exposed to diacetyl), 24 worms for Data Set 2.

To test this hypothesis, we first detected neurons whose activity profiles were correlated to the stimulus switch–we called them putative sensory neurons, since some interneurons are also known to be stimulus-correlated [20]. We then removed all interactions between these neurons, leaving only connections between putative sensory neurons to the rest of the network, and all interactions between non-sensory neurons (Fig 7G). We call this the Simplified Network, which included 98.4% and 98.2% of all edges in the full networks associated with Data Sets 1 and 2, respectively. Two of the six graph features modulated by stimulus identity in the Whole Network–namely, average and median weight–were also modulated in the Simplified Network (S15 Table). Furthermore, we achieved a higher accuracy using graph-theoretic features from the Simplified Network than was achieved using features of the Whole Network (S9 and S16 Tables). We next assessed whether these select features could improve our classifier’s generalizability.

We first tested whether a classifier trained on the features from one data set could perform well on the other data set. To this end, we removed all diacetyl sessions from Data Set 1 as they were not present in Data Set 2. Using the Whole Network, we found that training on Data Set 1 and testing on Data Set 2 led to moderate accuracies (Fig 7A, 33% accuracy); our accuracy was higher when we trained on Data Set 2 and tested on Data Set 1 (Fig 7B, 42% accuracy). Similarly, when we used the Simplified Network, we could achieve a moderate accuracy when we trained on Data Set 1 and tested on Data Set 2 (Fig 7C, 33% accuracy), and a higher accuracy in the other direction (Fig 7D, 42% accuracy). These results suggest that the classifier is detecting reliable patterns in the features computed on both the Whole and Simplified Networks.

We then combined Data Sets 1 and 2, and asked whether our classifier could accurately discriminate between the 4 chemical stimuli amongst the 48 worms. We hypothesized that there should be similar patterns in the features on both data sets, and that this would emerge in the Simplified Network, but not the Whole Network, because the latter would be “contaminated” by trivially-strong statistical interactions between putative sensory neurons. Moreover, this would allow us to determine whether the same patterns in the activity and graph-theoretic features appear in both data sets. Indeed, we found that a logistic regression classifier trained on Graph features from the Simplified Network performed much better at classifying stimulus identity than when trained on Graph features from the Whole Network (Fig 7E and 7F; 27% for Whole Network, 54% accuracy for Simplified Network). On the other hand, a logistic regression classifier trained on Activity features could not exceed chance classification accuracy (Fig 7E and 7F; 29% accuracy for both networks), indicating that the classifier might not learn patterns in the Activity features that are shared by both data sets. Overall, after removing strongly correlative interactions between putative sensory neurons, graph-theoretic features performed much better than activity features at decoding stimulus identity, suggesting that interactions amongst neurons better capture stimulus-modulated patterns in the network.

## Discussion

A central challenge in neuroscience is to develop tools that can uncover how stimulus properties, such as valence and identity, are encoded within neural networks. We monitored changes in the activity of over 50 neurons in the *C*. *elegans* head as the animal was exposed to diverse chemical stimuli. We found that activity statistics, such as the mean and standard deviation of neural activity and Fourier-based frequency measures, are not modulated by stimulus valence or identity; accordingly, they cannot be used to classify these stimulus properties in our combined data set of 48 worms. We then extracted graph-theoretic features from time-series activity traces of neurons and found that they were stimulus-dependent and improved our ability to predict stimulus identity, primarily when interactions between putative sensory neurons were removed. Importantly, these results were statistically robust and biologically reproducible when tested on two independently collected data sets. Our results suggest that some stimulus properties can be decoded using network features; this could be useful in instances where neuron labels are unknown or difficult to map across animals.

We used normalized mutual information to generate functional connectivity networks from neural activity traces. In contrast, recent work has tried to infer causal networks from activity traces using methods such as adaptive, sparsity-constrained Granger causality [51], network deconvolution [52], convex risk minimization [53], and convergent cross mapping [54]. Some of these methods, however, make assumptions about the data type and nature of causality that may not be true for our system (e.g., they assume spiking data as opposed to graded potentials, or they assume specific models of information spread, such as those used in epidemiology). While we used a relatively simple approach, our method is general and allows for any network inference algorithm to be plugged into our framework.

A major limitation of many previous methods is their propensity to infer interactions that are not real–apparent interactions driven by a third, unobserved party. We addressed this issue by removing interactions between neurons whose activity was closely correlated to the stimulus switch (i.e., putative sensory neurons). This improved our classifier’s ability to detect robust patterns in graph features from each data set–either independently, or in combination. More importantly, we provided evidence that interactions between a part of the network conventionally ignored in studies of sensory processing, namely the neurons not locked to stimulus onset or offset, are modulated by stimulus identity. By adapting our method, future studies of inferred neural interactions could examine the role played by a potentially understudied part of the nervous system in sensory processing.

The median network weight, transitivity, and max eigenvalue all emerged as useful features for discriminating stimulus identity. This implies that neural networks may use changes in the strength of interactions, particularly between local triplets of neurons, to facilitate communication and process stimulus identity. Accordingly, amongst the five stimuli tested, NaCl consistently induced the greatest increase in median weight, max eigenvalue, and transitivity at stimulus onset. This may reflect the nematode nervous system’s ability to use both salt and odor circuitry to encode sensory information when exposed to NaCl [17]. In contrast, the other stimuli that we used are primarily detected by olfactory circuits. Diacetyl, isoamyl alcohol, and 2-nonanone induced non-distinguishable increases in the max eigenvalue and transitivity of the neural network at stimulus onset. Benzaldehyde, on the other hand, evoked a change in these features that was intermediate to NaCl and the other three odorants. This suggests that the initial detection of an odorant may evoke changes in neural network organization that are then supplemented by stimulus-specific changes. Our classifier consistently detected NaCl and benzaldehyde most accurately, but tended to confuse the three other odorants (S14 Fig). Since the animal can behaviorally discriminate among these odorants [31], this suggests that the animal’s nervous system uses other methods to separate them.

In contrast to stimulus identity, stimulus valence did not significantly modulate any activity or graph feature. We suspect our mixed-effects model failed to detect differences between attractants and repellents because of the continuous nature of valence; some attractants are more attractive than others, and some repellents are more repellent than others. Thus, valence is not a binary variable with outcomes of ‘attractive’ or ‘repulsive,’ but a continuous variable more amenable to regression. Behavioral work that disentangles the relative valence of our chemical stimuli would be needed to build useful regression models. Furthermore, valence is intimately tied to concentration. For instance, some attractants can elicit repulsion at high concentrations. Since we used two concentrations per chemical, it is possible that we encountered a confound between concentration and valence that prevented us from finding a common representation of valence. Moreover, it may have been difficult to find a unifying representation when one of our repellents was an odorant (2-nonanone) and the other a tastant (NaCl). Consistent with this hypothesis, NaCl and 2-nonanone induced significantly different network organizations (Fig 4A, 4B and 4C), suggesting that chemicals of a similar valence can produce very different networks. Finally, work in other species has led to the hypothesis that valence may be encoded, partly, in specific subsets of neurons [55–58].

While we chose several graph-theoretic features to study diverse aspects of network organization [37,59], our list is not exhaustive and there may be other features that can better discriminate stimulus properties. We also focused on fully-connected weighted networks as opposed to sparse, binary networks that were formed by applying an arbitrary threshold to remove weak edges. Sparse network analysis depends on the precise threshold, which is difficult to set in a principled manner and may result in data that is difficult to interpret [60]. Attempts to use three different proportional thresholds to filter network weights consistently revealed that different features were significant depending on the threshold chosen (S3–S6 Tables), and the ability to classify stimulus identity was similarly variable (S10–S12 Tables). We focused on the simpler analysis of static networks–where a single network was generated around stimulus switches–because this technique is a new way of studying whole-brain neural activity. However, the network of interactions between neurons in any nervous system is dynamic (S8 Fig) and not the static entity we have inferred. Future work could refine this by studying multiple networks at different time-scales throughout stimulation. Finally, we used a basic machine learning approach to quantitatively test the power of using network features to classify stimulus properties. While we used sample sizes comparable to prior studies (n = 30 and 24 animals in Data Sets 1 and 2) and used standard approaches to avoid over-fitting (e.g., nested cross-validation, permutation testing), we nonetheless caution that building reliable machine learning models with relatively small data sets can be sensitive to a few data points. As it becomes experimentally easier to generate larger data sets with more neurons across many more conditions in other species, we anticipate being able to improve our framework of using graph-theoretic features of network activity to understand neural function.

## Materials and methods

### Ethics statement

All experiments in this manuscript have been approved by the Institutional Biosafety Committee at the Salk Institute for Biological Studies.

### Whole-brain calcium imaging

All imaging experiments were performed on a previously published strain (ZIM1048 *lite-1(ce314)X*, *msmIs4* [23]). We first trapped 30 young adults in a modified olfactory chip [19] that orients animals similarly [21]. Changes in GCaMP fluorescence were monitored using a Zeiss LSM 880 Airyscan (1.09–1.62 volumes/second) while the animal’s nose experienced buffer or one of five stimuli (Diacetyl 10^−4^%vol/vol, 10^−6^%vol/vol [61,62], benzaldehyde 10^−3^%vol/vol, 10^−6^%vol/vol [18], isoamyl alcohol 10^−5^%vol/vol, 10^−7^%vol/vol [20,63], 2-nonanone 10^−2^%vol/vol, 10^−4^%vol/vol [62] and NaCl at an aversive 600mM [32] and at an attractive 200mM [64] concentration). For each animal, we obtained a 21-minute recording with no stimulation (“Spontaneous”), a second 21-minute recording with M9 buffer changes (“Buffer”) and a third 21-minute recording with stimuli changes (“Stimulus”). Buffer and Stimulus sessions were interleaved for different animals except for those experiencing 2-nonanone, when Buffer always preceded Stimulus. The Stimulus or Buffer pattern was adapted from [30]. Example calcium-imaging videos of five worms experiencing one of each chemical are shown in S1–S5 Videos.

To test if our results were biologically reproducible, we recorded from the heads of another 24 young adults (i.e., Data Set 2) after analyzing the first 30 worms (i.e., Data Set 1). These 24 additional worms were exposed to one of four previously used stimuli (i.e., benzaldehyde, isoamyl alcohol, 2-nonanone, or NaCl) at the aforementioned concentrations (Fig 1C), for a total of 3 worms for each of 8 conditions (i.e., 4 stimuli times 2 concentrations each). We then focused our analyses on the same features we found to be significantly modulated on Data Set 1, thereby reducing the number of tests conducted on this second, independent data set (i.e., Data Set 2).

### Data processing

We first deconvolved the pixels using built-in Airyscan processing tools. We then corrected for motion using NormCorre (https://github.com/flatironinstitute/NoRMCorre) and extracted the raw fluorescence traces using CaImAn (https://github.com/flatironinstitute/CaImAn-MATLAB) [65–70]. We used multiple iterations using grids of decreasing size to register each video. 25 of 54 “Spontaneous” sessions had too much movement to correct, and were not further analyzed. To accurately identify all neurons in the head of the animal, we set the cutoff K, for the number of components to look for, to 140 (this is higher than the number of neurons previously detected in this strain [23]) allowing the CaImAn algorithm to detect many neurons with a high signal-to-noise ratio as recommended by [70]. Hence, neurons with no change in fluorescence were not detected. Finally, we used custom software to manually verify that regions of interest extracted by our analysis pipeline qualitatively matched the video, and to eliminate non-neuronal ROIs (e.g., gut granules).

### Measuring statistical features of neural activity

Each neuron’s activity trace was normalized within a session by the max fluorescence value reached in that 21-minute imaging session. To highlight the change in neural activity properties after a stimulus switch, we normalized the value of the property to its value pre-switch. For example, for stimulus onset, we computed the feature for every neuron in the first 30-seconds after stimulus onset and divided by the value of the feature in the 30-seconds prior to stimulus onset. We then took the average over all neurons. Thus, we report how much stimulus onset or offset changed the value of the feature from its pre-switch baseline to reflect how the addition or removal of a stimulus affected neural activity.

All analyses of the temporal dynamics of neural activity was performed using Fourier transforms. We used MATLAB’s periodogram to average the power of the four frequency bands used in our analyses (1^st^ band: 0.07–0.2 Hz, 2^nd^ band: 0.2–0.33 Hz, 3^rd^ band: 0.33–0.47 Hz, 4^th^ band: 0.47–0.6 Hz). We used MATLAB’s **spectrogram** to determine the max, average, and standard deviation of the frequency with the most power in a 30-second bin, using 10-second long sliding windows with 50% overlap. Thus, our measures quantified how much a switch from buffer to stimulus or stimulus to buffer changed activity.

### Determining functional connectivity

For each session, every neuron’s activity was normalized by the peak value it reached in the entire 21-minute imaging session, bounding every neuron’s activity in the range [0,1] (Fig 3A). Each session generated an *n* x *T* matrix, where *n* is the number of neurons and *T* is the length in time of imaging. Using this matrix, we generated two adjacency matrices, one for the pre-switch period and one for the post-switch period. Each of these periods lasted 30 seconds and resulted in 28 periods of activity: each session had 7 pulses, with 2 switches per pulse (i.e., onset and offset), and 2 pre- and post-switch periods per switch. We made two types of adjacency matrices for every block: one based on the absolute value of the Pearson’s correlation (PC), and the other based on the normalized mutual information (NMI) with a 0.05 bin size. The former was calculated using MATLAB’s built-in **corr.m** function. The latter was calculated per [43] as: $\mathrm{NMI}(A,B)=\frac{\mathrm{MI}(A,B)}{\sqrt{\mathrm{entropy}(A)\times \mathrm{entropy}(B)}}$, where entropy(A) is the entropy of the activity of neuron A, and MI(A,B) is the mutual information between the activity patterns of neurons A and B. We estimated the distribution of activity states for each neuron by counting the number of time points with activity levels that lie within one of ten equally-sized bins between 0 and 1 (i.e., 0–0.1, 0.1–0.2, 0.2–0.3, …, 0.9–1.0; Fig 3A and 3B). We then computed the entropy of each distribution, and the mutual information between each pair of neurons (Fig 3B and 3C). Finally, we computed the NMI between neurons A and B by using the entropy of each neuron and the mutual information between A and B (Fig 3D), as per the equation above. While many variants of NMI exist [71], the one we used has the desirable property of only reaching its maximum value when the distributions of states of two neurons are identical. If a neuron had an entropy of 0 because all its activity during the entire 30-second period was localized to one histogram bin (e.g., 0–0.05), then its edges to all other neurons were set to 0, effectively decoupling the neuron from the network; this only occurred for 1.3%, or 1,522 out of 116,676 cells across all imaging sessions and worms in Data Set 1, and 1.9%, or 1,622 out of 87,752 cells in Data Set 2. (Note: there are 30 worms in Data Set 1, and 24 worms in Data Set 2, hence the former has more cells.) We reasoned that a neuron whose activity did not appreciably change in a 30-second time period is not likely to be involved in processing of that stimulus. Finally, all self-loops were removed by setting the diagonal of the adjacency matrix to 0.

### Graph-theoretic analysis

Graph-theoretic features were calculated using the Brain Connectivity Toolbox [37,59] on the largest connected component of each network. For features that produced distributions, such as local efficiency, we report the mean value. We used the Louvain community detection algorithm to find modules [72]. The six features we found to be significantly modulated by stimulus identity on stimulus onset are:

1. Average weight = $\frac{1}{m}\sum_{i}w_{i},$

where *w*_*i*_ is the ith weight of the lower triangular portion of the weighted adjacency matrix *W*

1. Median weight = 50^th^ percentile of *w*_*i*_, where *w*_*i*_ is as defined for average weight
2. Max eigenvalue = *max*(*eig*(*W*)), where *W* is the weighted adjacency matrix
3. Average clustering coefficient = $\frac{1}{n}\sum_{i}c_{i}$, where *c*_*i*_ is the *i*th node’s clustering coefficient and *n* is the number of nodes in the network.
  - $c_{i}=\frac{1}{n}\sum_{i\in N}\frac{2t_{i}}{k_{i}(k_{i}-1)}$, where *t*_*i*_ is the geometric mean of the edge weights of the triangles around node i, *k*_*i*_ is the degree of node i, and *N* is the set of nodes in the network defined as follows:
  - $t_{i}=\frac{1}{2}\sum_{j,h\in N}{\left(w_{ij}w_{ih}w_{jh}\right)}^{1/3}$, where *w*_*ij*_ is the weight between nodes i and j
  - *k*_*i*_ = ∑_*j*∈*N*_ *w*_*ij*_
4. Transitivity = $\frac{\sum_{i\in N}2t_{i}}{\sum_{i\in N}k_{i}(k_{i}-1)}$
5. Average local efficiency = $\frac{1}{n}\sum_{i}l_{i}$, where *l*_*i*_ is the ith node’s local efficiency
  - $l_{i}=\frac{\sum_{j,h\in N;j\neq h}w_{ij}w_{ih}{\left[d_{jh}({\tilde{N}}_{i})\right]}^{-1}}{\sum_{j,h\in N;j\neq h}w_{ij}w_{ih}},$ where $d_{jh}({\tilde{N}}_{i})$ is the adapted shortest distance between nodes *j* and *h* in the network ${\tilde{N}}_{i}$ which contains all neighbors of node *i* excluding node *i* and after replacing *w*_*jh*_ to ${\tilde{w}}_{jh}$
  - $d_{jh}=\sum_{\begin{array}{c} k,l\\ a_{kl}\in g_{j↔h}^{W} \end{array}}\frac{1}{w_{kl}}$, where $g_{j↔h}^{W}$ is the shortest weighted path between nodes *j* and *h*
  - ${\tilde{w}}_{jh}=\frac{w_{jh}w_{ij}w_{ih}}{{\left[max(W)\right]}^{3}}$, where *W* is the weighted adjacency matrix

### Machine learning classification

The logistic regression classifier was built using one of three sets of features. The first set was composed of graph-theoretic features. The second set (called activity features) included statistical and temporal summaries of neural activity. The third set of features combined the first and second sets. All graph-theoretic features used to classify stimulus identity came from adjacency matrices constructed using NMI. A logistic regression classifier was trained on these three sets of features, and built using Python’s scikit-learn library using **LogisticRegressionCV** [73], with nested leave-one-out cross-validation to tune the regularization hyperparameter *C* and test generalization error; we tested values of C equal to 0.01, 0.1, 1, 10, and 100. Since we aimed to predict multiple classes (i.e., stimulus identity), we specifically used a multinomial logistic regression classifier (which we will call “logistic regression classifier”). The logistic regression classifier computes the probability that a set of input features belong to class *k*, where *k* is one of our chemical stimuli. It first computes the softmax score for each class as a weighted sum of input features, as follows:

$$s_{k}(x)={\left(\theta^{\left(k\right)}\right)}^{T}x.$$

The logistic regression classifier then estimates the probability that the given set of features belong to each class by passing the softmax scores through the softmax function, as follows:

$${\hat{p}}_{k}=\sigma {\left(s(x)\right)}_{k}=\frac{exp(s_{k}(x))}{\sum_{j=1}^{K}exp(s_{j}(x))},$$

and assigns the observation to the class with the largest probability.

Finally, to avoid over-fitting, we used permutation testing (Test 1 in [74]) in which we randomly permutated the labels of the classes 100 times and compared each classifier’s performance to its own null distribution. We took the relative position of the classifier’s true accuracy (trained on non-permutated labels) in this permutation distribution to be its p-value [74, 75]. This was a critical test that ensured our results were not merely due to chance, which is a significant yet often unappreciated issue in machine learning applications because theoretical chance is defined for sample sizes that are infinitely large [75].

### Statistical analyses for mixed-effects models

Our experiments consisted of multiple measurements from the same animal, grouped according to two ecologically-relevant aspects of chemical stimuli (i.e., stimulus valence or identity). This repeated-measures design is often modeled using a mixed-effects model because it accounts for: 1) fixed-effects owed to the ecologically-relevant condition (i.e., stimulus valence or identity), and 2) random-effects due to animal variability (e.g., some animals might naturally have a larger transitivity). Thus, we can provide a mixed-effects model, fit using MATLAB’s **fitglme.m**, with a design matrix that contains information on the time in seconds since the first pulse and an indicator variable denoting which stimulus property the animal experienced (e.g., attractant or repellent). A mixed-effects model that better explains the data using the class indicator than without it, tested using a likelihood ratio test (LRT, using **compare.m**), indicates a significant contribution from the fixed-effect to the model fit. To account for multiple comparisons of correlated features, we estimated the effective number of independent variables–m_eff_−and corrected the alpha of 0.05 to 0.05/m_eff_ [35]. The m_eff_ is computed as follows:

$$m_{eff}=1+(m-1)\left(1-\frac{var(\lambda )}{m}\right),$$

where *m* = the number of features (i.e., the 10 activity features or 24 graph features) and var(λ) is the variance of the eigenvalues of the correlation matrix of the features. The correction for Data Set 1 considered all measures together (i.e., graph and activity features), while the correction for Data Set 2 only considered those measures whose uncorrected p-value was less than 0.05 in Data Set 1; hence, m_eff_ was larger for Data Set 1 than Data Set 2. We then use pairwise F-tests using **coefTest.m** to assess which chemical stimuli lead to nervous system activity with significantly different means for feature *x*, provided that feature *x* has already passed the LRT.

We used a generalized linear mixed-effects model with a response distribution modeled as a gamma distribution. This allowed us to determine if a given feature was significantly modulated by stimulus identity or valence, while addressing the grouped nature of our data (i.e., multiple trials from individual worms which experienced different chemicals). We used the gamma distribution because it well-fit the distribution of most features’ values. We can write these models as the following linear regression:

y=Xβ+Zu+ε,

where **y** is an *N x 1* outcome vector (i.e., the value of the features we studied collected across animals and trials; for instance, *N =* 210 clustering coefficients if we had 30 animals and 7 trials for stimulus onset on Data Set 1, and *N* = 168 for Data Set 2 because we had 24 animals and 7 trials), **X** is the *N x p* design matrix of *p* predictor variables (i.e., it assigns observations to the right fixed effect level), **β** is a *p x 1* vector of fixed-effects regressions coefficients, **Z** is the *N x qJ* design matrix for the *q* random effects and *J* groups (i.e., it assigns observations to worms), **u** is a *qJ x 1* vector of *q* random effects for *J* groups, and **ε** is the *N x 1* vector of residuals (i.e., the part of **y** not explained by **Xβ + Zu**). Please see https://stats.idre.ucla.edu/other/mult-pkg/introduction-to-linear-mixed-models/ for more information.

We specified our null model in Wilkinson notation as:

y∼Pulse+(1|Animal),

where *y* is the feature’s values, *Pulse* is the time in seconds since the onset of the first stimulus pulse for each value of y, and *(1|Animal)* is a random effect for each animal. In our null model, *p* is 2, **X** is a two-column vector composed of a vector of ones for the fixed intercept and a vector of the values of *Pulse*, **β** is a vector of 2 coefficients, *q* is 1 because we only have 1 random intercept, and *J* is the number of animals (i.e., 30 in Data Set 1 and 24 in Data Set 2).

We specified our full model in Wilkinson notation as:

y∼StimulusCondition+Pulse+(1|Animal),

where *StimulusCondition* refers to a design matrix indicating which condition the animal experienced (i.e., either attractive or repellent for testing stimulus valence, or one of the chemical stimuli for testing stimulus identity). We can also write this equation as the aforementioned linear regression, with three changes: 1) *p* is 3 when we test valence (i.e., values of *Pulse*, column of ones for the fixed intercept, and a binary vector for the two levels of valence), 6 for testing identity on Data Set 1 (i.e., values of *Pulse*, the column of ones, and the 4 levels of chemical stimuli because one acts as the reference), and 5 for testing identity on Data Set 2 (because there are 3, not 4, levels of chemical stimuli since one acts as the reference); 2) **X** additionally assigns observations to the right stimulus condition; and 3) **β** is a vector of 3, 6, or 5 coefficients.

We then used the LRT to compute whether the full model (i.e., with *StimulusCondition*) fit the data better than the null model (i.e., only with *Pulse* and *(1|Animal)*). If the p-value was less than the m_eff_-corrected threshold, we conducted pairwise F-tests to assess which conditions are different from one another; the alpha value for the F-tests was adjusted by the number of possible pairwise comparisons in Data Set 1 (i.e., for the five chemical stimulus of Data Set 1, alpha = 0.05/10), and by the number of significant comparisons in Data Set 2 (i.e., alpha = 0.05/3, because NaCl was different from benzaldehyde, isoamyl alcohol, and 2-nonanone in Data Set 1).

## Supporting information

S1 Fig The same number of neurons were recorded during each stimulus trial. We observed similar numbers of neurons during experiments with any of the five chemicals in Data Set 1 (A) and four chemicals in Data Set 2 (B). p > 0.05 by Kruskal-Wallis test.
(TIF)

S2 Fig NaCl induces more variable activity on stimulus onset (Data Set 1). The first four features refer to mean, standard deviation, skewness, and kurtosis of neural activity. Power in 1st, 2nd, and 3rd bands refer to average power in the frequency ranges from 0.07–0.2 Hz, 0.2–0.34 Hz, and 0.34–0.47 Hz. Peak frequency is the frequency with the most power in a 30-second bin, and avg frequency and s.d. frequency are the average and standard deviation, respectively, of the frequencies with the most power in a sliding-window bin covering a 30-second period. Each color dot is the mean value across all seven pulses for a single worm, and the dark squares and lines indicate the mean and standard error of the mean across all worms. N = 21 for attractants and N = 9 for repellents (A), and N = 6 for each chemical stimulus (B). * p < 0.05 indicates features that were significant 1) by likelihood ratio test (LRT) on full and null generalized linear-mixed effects models (GLMEs), where the former included stimulus valence or identity as a fixed effect, and 2) by pairwise F-tests on stimulus coefficients of full GLME. Multiple comparisons correction for LRT used alpha = 0.05/meff, while those for F-tests used Bonferonni correction based on total number of distinct pairwise comparisons (i.e., alpha = 0.05/10).
(TIF)

S3 Fig Activity features are not modulated by stimulus valence or identity on stimulus offset (Data Set 1). The first four features refer to mean, standard deviation, skewness, and kurtosis of neural activity. Power in 1st, 2nd, and 3rd bands refer to average power in the frequency ranges from 0.07–0.2 Hz, 0.2–0.34 Hz, and 0.34–0.47 Hz. Peak frequency is the frequency with the most power in a 30-second bin, and avg frequency and s.d. frequency are the average and standard deviation, respectively, of the frequencies with the most power in a sliding-window bin covering a 30-second period. Each color dot is the mean value across all seven pulses for a single worm, and the dark squares and lines indicate the mean and standard error of the mean across all worms. N = 21 for attractants and N = 9 for repellents (A), and N = 6 for each chemical stimulus (B). p > 0.05 for all features by likelihood ratio test (LRT) on full and null generalized linear-mixed effects models, where the former included stimulus valence or identity as a fixed effect; as a result, no F-tests were used. Multiple comparisons correction for LRT used alpha = 0.05/meff.
(TIF)

S4 Fig Activity features are not modulated by stimulus valence or identity on stimulus onset (Data Set 2). The one significant feature in Data Set 1 is not significant in Data Set 2. The first four features refer to mean, standard deviation, skewness, and kurtosis of neural activity. Power in 1st, 2nd, and 3rd bands refer to average power in the frequency ranges from 0.07–0.2 Hz, 0.2–0.34 Hz, and 0.34–0.47 Hz. Peak frequency is the frequency with the most power in a 30-second bin, and avg frequency and s.d. frequency are the average and standard deviation, respectively, of the frequencies with the most power in a sliding-window bin covering a 30-second period. Each color dot is the mean value across all seven pulses for a single worm, and the dark squares and lines indicate the mean and standard error of the mean across all worms. N = 15 for attractants and N = 9 for repellents (A), and N = 6 for each chemical stimulus (B). p > 0.05 for all features by likelihood ratio test (LRT) on full and null generalized linear-mixed effects models, where the former included stimulus valence or identity as a fixed effect; as a result, no F-tests were used. Multiple comparisons correction for LRT used alpha = 0.05/meff.
(TIF)

S5 Fig Activity features are not modulated by stimulus valence or identity on stimulus offset (Data Set 2). The first four features refer to mean, standard deviation, skewness, and kurtosis of neural activity. Power in 1st, 2nd, and 3rd bands refer to average power in the frequency ranges from 0.07–0.2 Hz, 0.2–0.34 Hz, and 0.34–0.47 Hz. Peak frequency is the frequency with the most power in a 30-second bin, and avg frequency and s.d. frequency are the average and standard deviation, respectively, of the frequencies with the most power in a sliding-window bin covering a 30-second period. Each color dot is the mean value across all seven pulses for a single worm, and the dark squares and lines indicate the mean and standard error of the mean across all worms. N = 15 for attractants and N = 9 for repellents (A), and N = 6 for each chemical stimulus (B). p > 0.05 for all features by likelihood ratio test (LRT) on full and null generalized linear-mixed effects models, where the former included stimulus valence or identity as a fixed effect; as a result, no F-tests were used. Multiple comparisons correction for LRT used alpha = 0.05/meff.
(TIF)

S6 Fig Neural activity during stimulus sessions is higher-dimensional than during spontaneous sessions. Example neural dynamics observed during a Spontaneous session forms loops through principal components analysis space (A), but not during a Buffer (B) or Stimulus session (C). The percentages in panels A to C refer to percent of total variance explained by first three principal components (PCs). Generally, the first three PCs explain a larger percentage of the variance during Spontaneous sessions than during Stimulus sessions in both Data Set 1 (D) and 2 (E). The Stimulus sessions also have a larger participation ratio than the Spontaneous and Buffer sessions in Data Set 1 (F) and 2 (G). Kruskal-Wallis test, with Dunn-Sidak post-hoc test, * p < 0.05 (specifically, p = 0.04 and 0.01 for D and E, p = 0.0046 for F, and p = 0.0059 for Spont. vs Stimulus, and 0.03 for Buffer vs Stimulus in G).
(TIF)

S7 Fig Neural time series and adjacency matrices derived from S1–S5 Videos for five different worms, each exposed to one chemical. Ai, Bi, Ci, Di, and Ei depict all neurons recorded in five worms exposed to 2-nonanone (A), benzaldehyde (B), diacetyl (C), salt (D), or isoamyl alcohol (E). The time series were all normalized to have a maximum value of 1 by dividing each time series by its own maximum in the 21-minute long imaging session. Aii, Bii, Cii, Dii, and Eii depict the adjacency matrices computed from the time series by taking the normalized mutual information (NMI) between each pair of neurons; thus, for *n* neural time series, there is a corresponding *nxn* matrix of NMI values, with a minimum of 0 and maximum of 1. Note that in these examples we used the entire time series to compute the adjacency matrices, but in our analyses we used 30-second long periods of time.
(TIFF)

S8 Fig The neural networks of worms vary both within and across worms. The networks depicted here, for three example worms, show neurons (circles) connected by lines (edges), where the lines represent the normalized mutual information (NMI) between their time series. Only the strongest (i.e., NMI > = 0.4) interactions in the network are shown (hence, why many edges are missing), with those in green indicating the presence of a triangle (i.e., a triplet of connected neurons whose total weight > = 1.8) and those in blue its absence. All networks are based on neural activity observed in the 30-second period between 30 seconds and 1 minute into the beginning of either the Buffer (A, C, E) or Stimulus (B, D, F) session. Worms in panels A, C, and E were about to experience M9 buffer. Worms in panels B and D were about to experience benzaldehyde (BZ) at a concentration of 10e-6, while the worm in panel F was about to experience BZ at a concentration of 10–3. Some worms have more triangles prior to buffer onset (A) than stimulus onset (B), others have different numbers of connections that survive the 0.4 threshold (C, D), and others look similar (E, F).
(TIF)

S9 Fig Stimulus identity, but not valence, modulates graph features on stimulus onset (Data Set 1). Network features are derived from adjacency matrices constructed with normalized mutual information. See S1 Table for description of features. Each color dot is the mean value across all seven pulses for a single worm, and the dark squares and lines indicate the mean and standard error of the mean across all worms. N = 21 for attractants and N = 9 for repellents (A), and N = 6 for each chemical stimulus (B). * p < 0.05 indicates features that were significant 1) by likelihood ratio test (LRT) on full and null generalized linear-mixed effects models (GLMEs), where the former included stimulus valence or identity as a fixed effect, and 2) by pairwise F-tests on stimulus coefficients of full GLME. Multiple comparisons correction for LRT used alpha = 0.05/meff, while those for F-tests used Bonferonni correction based on total number of distinct pairwise comparisons (i.e., alpha = 0.05/10).
(TIF)

S10 Fig Graph features are not modulated by stimulus valence or identity on stimulus offset (Data Set 1). Network features are derived from adjacency matrices constructed with normalized mutual information. See S1 Table for description of features. Each color dot is the mean value across all seven pulses for a single worm, and the dark squares and lines indicate the mean and standard error of the mean across all worms. N = 21 for attractants and N = 9 for repellents (A), and N = 6 for each chemical stimulus (B). p > 0.05 for all features by likelihood ratio test (LRT) on full and null generalized linear-mixed effects models, where the former included stimulus valence or identity as a fixed effect; as a result, no F-tests were used. Multiple comparisons correction for LRT used alpha = 0.05/meff.
(TIF)

S11 Fig Stimulus identity, but not valence, modulates graph features on stimulus onset (Data Set 2). Six of the seven significant features from Data Set 1 are also significant in Data Set 2. Network features are derived from adjacency matrices constructed with normalized mutual information. See S1 Table for description of features. Each color dot is the mean value across all seven pulses for a single worm, and the dark squares and lines indicate the mean and standard error of the mean across all worms. N = 15 for attractants and N = 9 for repellents (A), and N = 6 for each chemical stimulus (B). * p < 0.05 indicates features that were significant 1) by likelihood ratio test (LRT) on full and null generalized linear-mixed effects models (GLMEs), where the former included stimulus valence or identity as a fixed effect, and 2) by pairwise F-tests on stimulus coefficients of full GLME. Multiple comparisons correction for LRT used alpha = 0.05/meff, while those for F-tests used Bonferonni correction based on total number of distinct pairwise comparisons (i.e., alpha = 0.05/3).
(TIF)

S12 Fig Graph features are not modulated by stimulus valence or identity on stimulus offset (Data Set 2). Network features are derived from adjacency matrices constructed with normalized mutual information. See S1 Table for description of features. Each color dot is the mean value across all seven pulses for a single worm, and the dark squares and lines indicate the mean and standard error of the mean across all worms. N = 15 for attractants and N = 9 for repellents (A), and N = 6 for each chemical stimulus (B). p > 0.05 for all features by likelihood ratio test (LRT) on full and null generalized linear-mixed effects models, where the former included stimulus valence or identity as a fixed effect; as a result, no F-tests were used. Multiple comparisons correction for LRT used alpha = 0.05/meff.
(TIF)

S13 Fig Trimming Data Set 1 to the same size as Data Set 2 improves classification accuracy of stimulus identity using graph features alone. A Logistic Regression classifier was trained to identify chemical stimulus using nested leave-one-out cross-validation on Data Set 1 with all six diacetyl sessions removed for stimulus onset (A, C) and offset (B, D). We used Activity features (light blue bars), Graph features (dark blue bars), and both Activity and Graph features together (green bars). The classifier achieved statistically significant above-chance accuracy on stimulus onset and offset with Graph features alone (p = 0.01 and 0.03 for panels A and B), which was higher than in the full Data Set 1. The dashed red line refers to chance accuracy, which is 25% because Data Set 1 was trimmed to the 4 chemical stimuli studied in Data Set 2 (i.e., benzaldehyde, isoamyl alcohol, 2-nonanone, and NaCl). Statistical significance was assessed using a permutation test with n = 100 permutations. N = 24 worms. * refer to classifier with accuracy significantly above chance according to permutation test.
(TIF)

S14 Fig Confusion matrices for stimulus classification on Data Sets 1 and 2 using three sets of features. We trained a Logistic Regression classifier with nested leave-one-out cross-validation based on either activity (A, D), graph (B, E), or combined (C, F) features for the Whole Network on Data Sets 1 (DS1; A—C) and 2 (DS2; D—F). The true chemicals are labeled on the rows, and the predicted chemicals on the columns. Chemicals along the main diagonal are correct predictions. The chemicals are benzaldehyde (B), diacetyl (D), isoamyl alcohol (I), 2-nona- none (N), and NaCl (S, for salt). Since there are six animals per condition, the largest possible value is 6, with darker squares indicating more predictions of that column’s label. Note that B and S are often classified best (i.e., have darker squares on the main diagonal) using graph features. All features were standardized. N = 30 worms for Data Set 1, 24 worms for Data Set 2, and 6 worms per chemical.
(TIF)

S1 Table Definition of graph-theoretic features studied in this manuscript. The twenty-two listed features are grouped into one of five classes: basic structure (of the adjacency matrix), functional segregation (measures of the decomposability of the network), functional integration (the potential for disparate parts of the network to communicate), centrality (the importance of any one neuron to network communication), and resilience to perturbations, such as lesions (measures of how robust the system is to disruptions at individual nodes). For a review, see (1).
(DOCX)

S2 Table Graph-theoretic results from likelihood ratio test on generalized linear mixed-effects models. Results from the likelihood ratio test applied on a full vs null model. The null model includes information on animal ID and time since first pulse. The full model includes information on either Valence or Identity in addition to the null model. The p-values in red indicate a significant difference in the data’s likelihood when explained with the full model vs the null model; hence, the parameter (i.e., Valence or Identity) significantly improved model fit. DS1 and DS2 refers to Data Sets 1 and 2. The p-values in bold red indicate a significant difference in DS1 and DS2. The m_eff_ for Identity comparison on Buffer Onset, Buffer Offset, Stimulus Onset, and Stimulus Offset were 28.1, 27.8, 27.1, and 28.0 for DS1, and 1.0, 2.8, 6.2, and 8.9 for DS2. (Note that m_eff_ for DS2 is computed solely on the features that had p-values less than 0.05 in DS1; hence, it is smaller in DS2.)
(DOCX)

S3 Table Graph-theoretic results from likelihood ratio test on generalized linear mixed-effects models for the Buffer session on thresholded networks for DS1. Only the top 30%, 20%, or 10% of weights were kept in each network (columns). Results from the likelihood ratio test applied on a full vs null model. The null model includes information on animal ID and time since first pulse. The full model includes information on either Valence or Identity in addition to the null model. The p-values in red indicate a significant difference in the data’s likelihood when explained with the full model vs the null model; hence, the parameter (i.e., Valence or Identity) significantly improved model fit. DS1 and DS2 refers to Data Sets 1 and 2. The p-values in bold red indicate a significant difference in DS1 and DS2. Any missing entries did not converge. The m_eff_ for Identity comparison on Buffer Onset and Buffer Offset were, for 30%, 28.3, 26.4; for 20%, 27.3, 27.0; for 10%, 27.5, 28.0.
(DOCX)

S4 Table Graph-theoretic results from likelihood ratio test on generalized linear mixed-effects models for the Stimulus session on thresholded networks for DS1. Only the top 30%, 20%, or 10% of weights were kept in each network (columns). Results from the likelihood ratio test applied on a full vs null model. The null model includes information on animal ID and time since first pulse. The full model includes information on either Valence or Identity in addition to the null model. The p-values in red indicate a significant difference in the data’s likelihood when explained with the full model vs the null model; hence, the parameter (i.e., Valence or Identity) significantly improved model fit. DS1 and DS2 refers to Data Sets 1 and 2. The p-values in bold red indicate a significant difference in DS1 and DS2. Any missing entries did not converge. The m_eff_ for Identity comparison on Stimulus Onset and Stimulus Offset were, for 30%, 26.3, 27.2; for 20%, 26.4, 27.4; for 10%, 26.7, 27.3.
(DOCX)

S5 Table Graph-theoretic results from likelihood ratio test on generalized linear mixed-effects models for the Buffer session on thresholded networks for DS2. Only the top 30%, 20%, or 10% of weights were kept in each network (columns). Results from the likelihood ratio test applied on a full vs null model. The null model includes information on animal ID and time since first pulse. The full model includes information on either Valence or Identity in addition to the null model. The p-values in red indicate a significant difference in the data’s likelihood when explained with the full model vs the null model; hence, the parameter (i.e., Valence or Identity) significantly improved model fit. DS1 and DS2 refers to Data Sets 1 and 2. The p-values in bold red indicate a significant difference in DS1 and DS2. Any missing entries did not converge. The m_eff_ for Identity comparison on Buffer Onset and Buffer Offset were, for 30%, 0, 10.5; for 20%, 1, 9.9; for 10%, 0, 11.7.
(DOCX)

S6 Table Graph-theoretic results from likelihood ratio test on generalized linear mixed-effects models for the Stimulus session on thresholded networks for DS2. Only the top 30%, 20%, or 10% of weights were kept in each network (columns). Results from the likelihood ratio test applied on a full vs null model. The null model includes information on animal ID and time since first pulse. The full model includes information on either Valence or Identity in addition to the null model. The p-values in red indicate a significant difference in the data’s likelihood when explained with the full model vs the null model; hence, the parameter (i.e., Valence or Identity) significantly improved model fit. DS1 and DS2 refers to Data Sets 1 and 2. The p-values in bold red indicate a significant difference in DS1 and DS2. Any missing entries did not converge. The m_eff_ for Identity comparison on Stimulus Onset and Stimulus Offset were, for 30%, 11.9, 11.2; for 20%, 9.6, 8.0; for 10%, 10.5, 6.8.
(DOCX)

S7 Table Graph-theoretic results from likelihood ratio test on generalized linear mixed-effects models for the Buffer session on differently correlated networks. The positively (+) and negatively (-) correlated subnetworks are shown (columns). Results from the likelihood ratio test applied on a full vs null model. The null model includes information on animal ID and time since first pulse. The full model includes information on either Valence or Identity in addition to the null model. The p-values in red indicate a significant difference in the data’s likelihood when explained with the full model vs the null model; hence, the parameter (i.e., Valence or Identity) significantly improved model fit. DS1 and DS2 refers to Data Sets 1 and 2. The p-values in bold red indicate a significant difference in DS1 and DS2. Any missing entries did not converge. The m_eff_ for Identity comparison on Onset (+), Onset (-), Offset (+), and Offset (-) were 28.9, 29.2, 27.0, and 27.2 for DS1, and 2.0, 1.0, 5.5, and 4.7 for DS2. (Note that m_eff_ for DS2 is computed solely on the features that had p-values less than 0.05 in DS1; hence, it is smaller in DS2.)
(DOCX)

S8 Table Graph-theoretic results from likelihood ratio test on generalized linear mixed-effects models for the Stimulus session on differently correlated networks. The positively (+) and negatively (-) correlated subnetworks are shown (columns). Results from the likelihood ratio test applied on a full vs null model. The null model includes information on animal ID and time since first pulse. The full model includes information on either Valence or Identity in addition to the null model. The p-values in red indicate a significant difference in the data’s likelihood when explained with the full model vs the null model; hence, the parameter (i.e., Valence or Identity) significantly improved model fit. DS1 and DS2 refers to Data Sets 1 and 2. The p-values in bold red indicate a significant difference in DS1 and DS2. Any missing entries did not converge. The m_eff_ for Identity comparison on Onset (+), Onset (-), Offset (+), and Offset (-) were 27.4, 29.2, 28.0, and 29.2 for DS1, and 9.8, 9.2, 6.6, and 6.5 for DS2. (Note that m_eff_ for DS2 is computed solely on the features that had p-values less than 0.05 in DS1; hence, it is smaller in DS2.)
(DOCX)

S9 Table Classifier results from permutation testing on full networks. Best performance achieved by logistic regression classifier on a specific classification task–namely, for a given session, pulse switch type, and one of three sets of features, correctly classify responses. The nested leave-one-out cross validation accuracy, the mean and standard deviation of the accuracies of a null distribution built using 100 permutations of the labels, and the corresponding p-value, or relative position of its accuracy in the null distribution, are all listed. Values in red attained significantly above-chance accuracies, and those in bold red did so in Data Sets 1 and 2. Some tasks did not exceed chance (e.g., stimulus onset during Buffer sessions on Data Set 1), and this is indicated by a dashed line to indicate that no permutation testing was conducted. Accuracy on some classification tasks was higher when features were standardized (y = yes, n = no, y/n = same accuracy with or without standardization). Chance is 20% for Data Set 1 (DS1) and 25% for Data Set (DS2). GT = Graph Theory, Comb = Activity + Graph Theory.
(DOCX)

S10 Table Classifier results from permutation testing on networks with the top 30% of weights. Best performance achieved by logistic regression classifier on a specific classification task–namely, for a given session, pulse switch type, and one of three sets of features, correctly classify responses. The nested leave-one-out cross validation accuracy, the mean and standard deviation of the accuracies of a null distribution built using 100 permutations of the labels, and the corresponding p-value, or relative position of its accuracy in the null distribution, are all listed. Values in red attained significantly above-chance accuracies, and those in bold red did so in Data Sets 1 and 2. Some tasks did not exceed chance (e.g., stimulus onset during Buffer sessions on Data Set 1), and this is indicated by a dashed line to indicate that no permutation testing was conducted. Accuracy on some classification tasks was higher when features were standardized (y = yes, n = no, y/n = same accuracy with or without standardization). Chance is 20% for Data Set 1 (DS1) and 25% for Data Set (DS2). GT = Graph Theory, Comb = Activity + Graph Theory.
(DOCX)

S11 Table Classifier results from permutation testing on networks with the top 20% of weights. Best performance achieved by logistic regression classifier on a specific classification task–namely, for a given session, pulse switch type, and one of three sets of features, correctly classify responses. The nested leave-one-out cross validation accuracy, the mean and standard deviation of the accuracies of a null distribution built using 100 permutations of the labels, and the corresponding p-value, or relative position of its accuracy in the null distribution, are all listed. Values in red attained significantly above-chance accuracies, and those in bold red did so in Data Sets 1 and 2. Some tasks did not exceed chance (e.g., stimulus onset during Buffer sessions on Data Set 2), and this is indicated by a dashed line to indicate that no permutation testing was conducted. Accuracy on some classification tasks was higher when features were standardized (y = yes, n = no, y/n = same accuracy with or without standardization). Chance is 20% for Data Set 1 (DS1) and 25% for Data Set (DS2). GT = Graph Theory, Comb = Activity + Graph Theory.
(DOCX)

S12 Table Classifier results from permutation testing on networks with the top 10% of weights. Best performance achieved by logistic regression classifier on a specific classification task–namely, for a given session, pulse switch type, and one of three sets of features, correctly classify responses. The nested leave-one-out cross validation accuracy, the mean and standard deviation of the accuracies of a null distribution built using 100 permutations of the labels, and the corresponding p-value, or relative position of its accuracy in the null distribution, are all listed. Values in red attained significantly above-chance accuracies, and those in **bold red** did so in Data Sets 1 and 2. Some tasks did not exceed chance (e.g., stimulus onset during Buffer sessions on Data Set 1), and this is indicated by a dashed line to indicate that no permutation testing was conducted. Accuracy on some classification tasks was higher when features were standardized (y = yes, n = no, y/n = same accuracy with or without standardization). Chance is 20% for Data Set 1 (DS1) and 25% for Data Set (DS2). GT = Graph Theory, Comb = Activity + Graph Theory.
(DOCX)

S13 Table Classifier results from permutation testing on networks with positively correlated neurons. Best performance achieved by logistic regression classifier on a specific classification task–namely, for a given session, pulse switch type, and one of three sets of features, correctly classify responses. The nested leave-one-out cross validation accuracy, the mean and standard deviation of the accuracies of a null distribution built using 100 permutations of the labels, and the corresponding p-value, or relative position of its accuracy in the null distribution, are all listed. Values in red attained significantly above-chance accuracies, and those in bold red did so in Data Sets 1 and 2. Some tasks did not exceed chance (e.g., stimulus onset during Buffer sessions on Data Set 1), and this is indicated by a dashed line to indicate that no permutation testing was conducted. Accuracy on some classification tasks was higher when features were standardized (y = yes, n = no, y/n = same accuracy with or without standardization). Chance is 20% for Data Set 1 (DS1) and 25% for Data Set (DS2). GT = Graph Theory, Comb = Activity + Graph Theory.
(DOCX)

S14 Table Classifier results from permutation testing on networks with negatively correlated neurons. Best performance achieved by logistic regression classifier on a specific classification task–namely, for a given session, pulse switch type, and one of three sets of features, correctly classify responses. The nested leave-one-out cross validation accuracy, the mean and standard deviation of the accuracies of a null distribution built using 100 permutations of the labels, and the corresponding p-value, or relative position of its accuracy in the null distribution, are all listed. Values in red attained significantly above-chance accuracies, and those in bold red did so in Data Sets 1 and 2. Some tasks did not exceed chance (e.g., stimulus onset during Buffer sessions on Data Set 1), and this is indicated by a dashed line to indicate that no permutation testing was conducted. Accuracy on some classification tasks was higher when features were standardized (y = yes, n = no, y/n = same accuracy with or without standardization). Chance is 20% for Data Set 1 (DS1) and 25% for Data Set (DS2). GT = Graph Theory, Comb = Activity + Graph Theory.
(DOCX)

S15 Table Graph-theoretic results from likelihood ratio test on generalized linear mixed-effects models for Simplified Network. Results from the likelihood ratio test applied on a full vs null model. The null model includes information on animal ID and time since first pulse. The full model includes information on either Valence or Identity in addition to the null model. The p-values in red indicate a significant difference in the data’s likelihood when explained with the full model vs the null model; hence, the parameter (i.e., Valence or Identity) significantly improved model fit. DS1 and DS2 refers to Data Sets 1 and 2. The p-values in bold red indicate a significant difference in DS1 and DS2. Any missing entries did not converge. The m_eff_ for Identity comparison on Buffer Onset, Buffer Offset, Stimulus Onset, and Stimulus Offset were 17.3, 18.5, 18.8, and 17.8 for DS1, and 2.0, 1.4, 6.0, and 5.2 for DS2. (Note that m_eff_ for DS2 is computed solely on the features that had p-values less than 0.05 in DS1; hence, it is smaller in DS2.)
(DOCX)

S16 Table Classifier results from permutation testing on Simplified Network. Performance achieved by logistic regression classifier on a specific classification task–namely, for a given session, pulse switch type, and one of three sets of features, correctly classify responses. The nested leave-one-out cross validation accuracy, the mean and standard deviation of the accuracies of a null distribution built using 100 permutations of the labels, and the corresponding p-value, or relative position of its accuracy in the null distribution, are all listed. We only used non-standardized graph features computed on the Simplified Network. Values in red attained significantly above-chance accuracies, and those in bold red did so in Data Sets 1 and 2. Some tasks did not exceed chance (e.g., stimulus onset during Buffer sessions on Data Set 1), and this is indicated by a dashed line to indicate that no permutation testing was conducted. Chance is 20% for Data Set 1 (DS1) and 25% for Data Set (DS2).
(DOCX)

S17 Table Sensitivity analysis of statistically significant results: dependence on time bin. Results from the likelihood ratio test applied on a full vs null model. The null model includes information on animal ID and time since first pulse. The full model includes information on Identity in addition to the null model. The p-values in red indicate a significant difference in the data’s likelihood when explained with the full model vs the null model; hence, the parameter (i.e., Identity) significantly improved model fit. DS1 and DS2 refers to Data Sets 1 and 2. The p-values in bold red indicate a significant difference in DS1 and DS2. All networks formed with NMI bin 0.05, as in the main text. Features shown are for stimulus onset. The m_eff_ for 15, 23, and 30 seconds was 29, 27.6, 27.1 for DS1, and 15.5, 10.3, 6.2 for DS2.
(DOCX)

S18 Table Sensitivity analysis of statistically non-significant results: dependence on time bin. Results from the likelihood ratio test applied on a full vs null model. The null model includes information on animal ID and time since first pulse. The full model includes information on Identity in addition to the null model. The p-values in red indicate a significant difference in the data’s likelihood when explained with the full model vs the null model; hence, the parameter (i.e., Identity) significantly improved model fit. DS1 and DS2 refers to Data Sets 1 and 2. The p-values in bold red indicate a significant difference in DS1 and DS2. All networks formed with NMI bin 0.05, as in the main text. Features shown are for stimulus onset. The m_eff_ for 15, 23, and 30 seconds was 29, 27.6, 27.1 for DS1, and 15.5, 10.3, 6.2 for DS2.
(DOCX)

S19 Table Sensitivity analysis of statistically significant results: dependence on NMI bin. Results from the likelihood ratio test applied on a full vs null model. The null model includes information on animal ID and time since first pulse. The full model includes information on Identity in addition to the null model. The p-values in red indicate a significant difference in the data’s likelihood when explained with the full model vs the null model; hence, the parameter (i.e., Identity) significantly improved model fit. DS1 and DS2 refers to Data Sets 1 and 2. The p-values in bold red indicate a significant difference in DS1 and DS2. All networks formed with time bin 30 seconds, as in the main text. Features shown are for stimulus onset. The m_eff_ for 0.05, 0.1, and 0.2 bins was 27.1, 27.4, 25.5 for DS1, and 6.2, 13.6, 9.9 for DS2.
(DOCX)

S20 Table Sensitivity analysis of statistically non-significant results: dependence on NMI bin. Results from the likelihood ratio test applied on a full vs null model. The null model includes information on animal ID and time since first pulse. The full model includes information on Identity in addition to the null model. The p-values in red indicate a significant difference in the data’s likelihood when explained with the full model vs the null model; hence, the parameter (i.e., Identity) significantly improved model fit. DS1 and DS2 refers to Data Sets 1 and 2. The p-values in bold red indicate a significant difference in DS1 and DS2. All networks formed with time bin 30 seconds, as in the main text. Features shown are for stimulus onset. The meff for 0.05, 0.1, and 0.2 bins was 27.1, 27.4, 25.5 for DS1, and 6.2, 13.6, 9.9 for DS2.
(DOCX)

S21 Table Sensitivity analysis of classifier results for Data Set 1: dependence on time bin and NMI bin. Performance achieved by logistic regression classifier on a specific classification task–namely, correctly classify responses based on the graph features computed for the first pulse of a stimulus session given a certain time bin and NMI bin. The nested leave-one-out cross validation accuracy, the mean and standard deviation of the accuracies of a null distribution built using 100 permutations of the labels, and the corresponding p-value, or relative position of its accuracy in the null distribution, are all listed. We only used non-standardized graph features computed on the Whole Network. Values in red attained significantly above-chance accuracies, and those in bold red did so in Data Sets 1 and 2. Some tasks did not exceed chance (e.g., time bin 15 x NMI bin 0.05), and this is indicated by a dashed line to indicate that no permutation testing was conducted. Chance is 20%.
(DOCX)

S22 Table Sensitivity analysis of classifier results for Data Set 2: dependence on time bin and NMI bin. Performance achieved by logistic regression classifier on a specific classification task–namely, correctly classify responses based on the graph features computed for the first pulse of a stimulus session given a certain time bin and NMI bin. The nested leave-one-out cross validation accuracy, the mean and standard deviation of the accuracies of a null distribution built using 100 permutations of the labels, and the corresponding p-value, or relative position of its accuracy in the null distribution, are all listed. We only used non-standardized graph features computed on the Whole Network. Values in red attained significantly above-chance accuracies, and those in bold red did so in Data Sets 1 and 2. Some tasks did not exceed chance (e.g., time bin 15 x NMI bin 0.1), and this is indicated by a dashed line to indicate that no permutation testing was conducted. Chance is 25%.
(DOCX)

S23 Table Weight vectors learned by logistic regression classifier trained on Whole Network, Data Set 1, using activity features. B, D, I, N, and S refer to benzaldehyde, diacetyl, isoamyl alcohol, 2-nonanone, and NaCl (salt). The weights are log odds; hence, an unit increase in that row’s feature increases or decreases the log odds of the model predicting that the sample belongs to that column’s stimulus. The features with the largest positive and negative weights for each stimulus are colored red, and those found to be significantly modulated by stimulus identity via our mixed-effects model are highlighted. The features were standardized as this gave the best performance for the classifier (see S9 Table).
(DOCX)

S24 Table Weight vectors learned by logistic regression classifier trained on Whole Network, Data Set 1, using graph features. B, D, I, N, and S refer to benzaldehyde, diacetyl, isoamyl alcohol, 2-nonanone, and NaCl (salt). The weights are log odds; hence, an unit increase in that row’s feature increases or decreases the log odds of the model predicting that the sample belongs to that column’s stimulus. The features with the largest positive and negative weights for each stimulus are colored red, and those found to be significantly modulated by stimulus identity via our mixed-effects model are highlighted. The features were not standardized as this gave the best performance for the classifier (see S9 Table).
(DOCX)

S25 Table Weight vectors learned by logistic regression classifier trained on Whole Network, Data Set 1, using activity and graph features. B, D, I, N, and S refer to benzaldehyde, diacetyl, isoamyl alcohol, 2-nonanone, and NaCl (salt). The weights are log odds; hence, an unit increase in that row’s feature increases or decreases the log odds of the model predicting that the sample belongs to that column’s stimulus. The prefixes ‘Graph’ and ‘Activity’ indicate whether the feature is a graph or activity feature. The features with the largest positive and negative weights for each stimulus are colored red, and those found to be significantly modulated by stimulus identity via our mixed-effects model are highlighted. The features were not standardized as this gave the best performance for the classifier (see S9 Table).
(DOCX)

S26 Table Weight vectors learned by logistic regression classifier trained on Whole Network, Data Set 2, using activity features. B, D, I, N, and S refer to benzaldehyde, diacetyl, isoamyl alcohol, 2-nonanone, and NaCl (salt). The weights are log odds; hence, an unit increase in that row’s feature increases or decreases the log odds of the model predicting that the sample belongs to that column’s stimulus. The features with the largest positive and negative weights for each stimulus are colored red, and those found to be significantly modulated by stimulus identity via our mixed-effects model are highlighted. The features were standardized as this gave the best performance for the classifier (see S9 Table).
(DOCX)

S27 Table Weight vectors learned by logistic regression classifier trained on Whole Network, Data Set 2, using graph features. B, D, I, N, and S refer to benzaldehyde, diacetyl, isoamyl alcohol, 2-nonanone, and NaCl (salt). The weights are log odds; hence, an unit increase in that row’s feature increases or decreases the log odds of the model predicting that the sample belongs to that column’s stimulus. The features with the largest positive and negative weights for each stimulus are colored red, and those found to be significantly modulated by stimulus identity via our mixed-effects model are highlighted. The features were not standardized as this gave the best performance for the classifier (see S9 Table).
(DOCX)

S28 Table Weight vectors learned by logistic regression classifier trained on Whole Network, Data Set 2, using activity and graph features. B, D, I, N, and S refer to benzaldehyde, diacetyl, isoamyl alcohol, 2-nonanone, and NaCl (salt). The weights are log odds; hence, an unit increase in that row’s feature increases or decreases the log odds of the model predicting that the sample belongs to that column’s stimulus. The prefixes ‘Graph’ and ‘Activity’ indicate whether the feature is a graph or activity feature. The features with the largest positive and negative weights for each stimulus are colored red, and those found to be significantly modulated by stimulus identity via our mixed-effects model are highlighted. The features were standardized as this gave the best performance for the classifier (see S9 Table).
(DOCX)

S1 Video One worm experiences 2-nonanone over the course of 21 minutes. The stimulus is present when the white square appears in the top right of the video, and is schematically represented in Fig 1B. The neural traces extracted by CaImAn and corresponding adjacency matrix are shown in S7A and S7B Fig. We created this file.
(MP4)

S2 Video One worm experiences benzaldehyde over the course of 21 minutes. The stimulus is present when the white square appears in the top right of the video, and is schematically represented in Fig 1B. The neural traces extracted by CaImAn and corresponding adjacency matrix are shown in S7C and S7D Fig. We created this file.
(MP4)

S3 Video One worm experiences diacetyl over the course of 21 minutes. The stimulus is present when the white square appears in the top right of the video, and is schematically represented in Fig 1B. The neural traces extracted by CaImAn and corresponding adjacency matrix are shown in S7E and S7F Fig. We created this file.
(MP4)

S4 Video One worm experiences NaCl over the course of 21 minutes. The stimulus is present when the white square appears in the top right of the video, and is schematically represented in Fig 1B. The neural traces extracted by CaImAn and corresponding adjacency matrix are shown in S7G and S7H Fig. We created this file.
(MP4)

S5 Video One worm experiences isoamyl alcohol over the course of 21 minutes. The stimulus is present when the white square appears in the top right of the video, and is schematically represented in Fig 1B. The neural traces extracted by CaImAn and corresponding adjacency matrix are shown in S7I and S7J Fig. We created this file.
(MP4)
