# Supplementary figures and images for: Neural network features distinguish chemosensory stimuli in Caenorhabditis elegans
Source: PLoS Comput Biol. 2021 Nov 9;17(11):e1009591. doi: 10.1371/journal.pcbi.1009591 (PMC8604368; doi:10.1371/journal.pcbi.1009591)

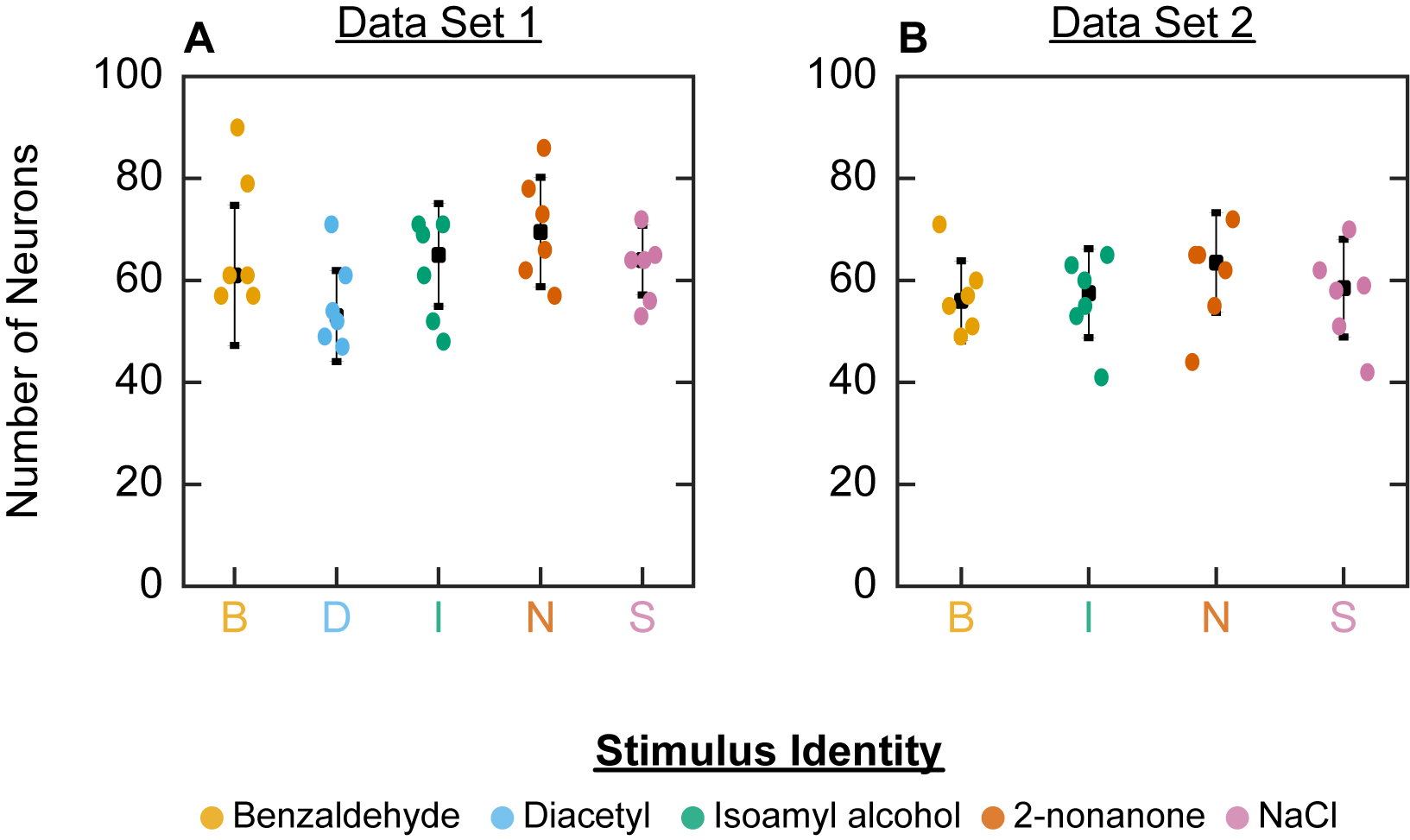

Supplement: S1 Fig — We observed similar numbers of neurons during experiments with any of the five chemicals in Data Set 1 (A) and four chemicals in Data Set 2 (B). p > 0.05 by Kruskal-Wallis test. (TIF) [file pcbi.1009591.s001.tif]

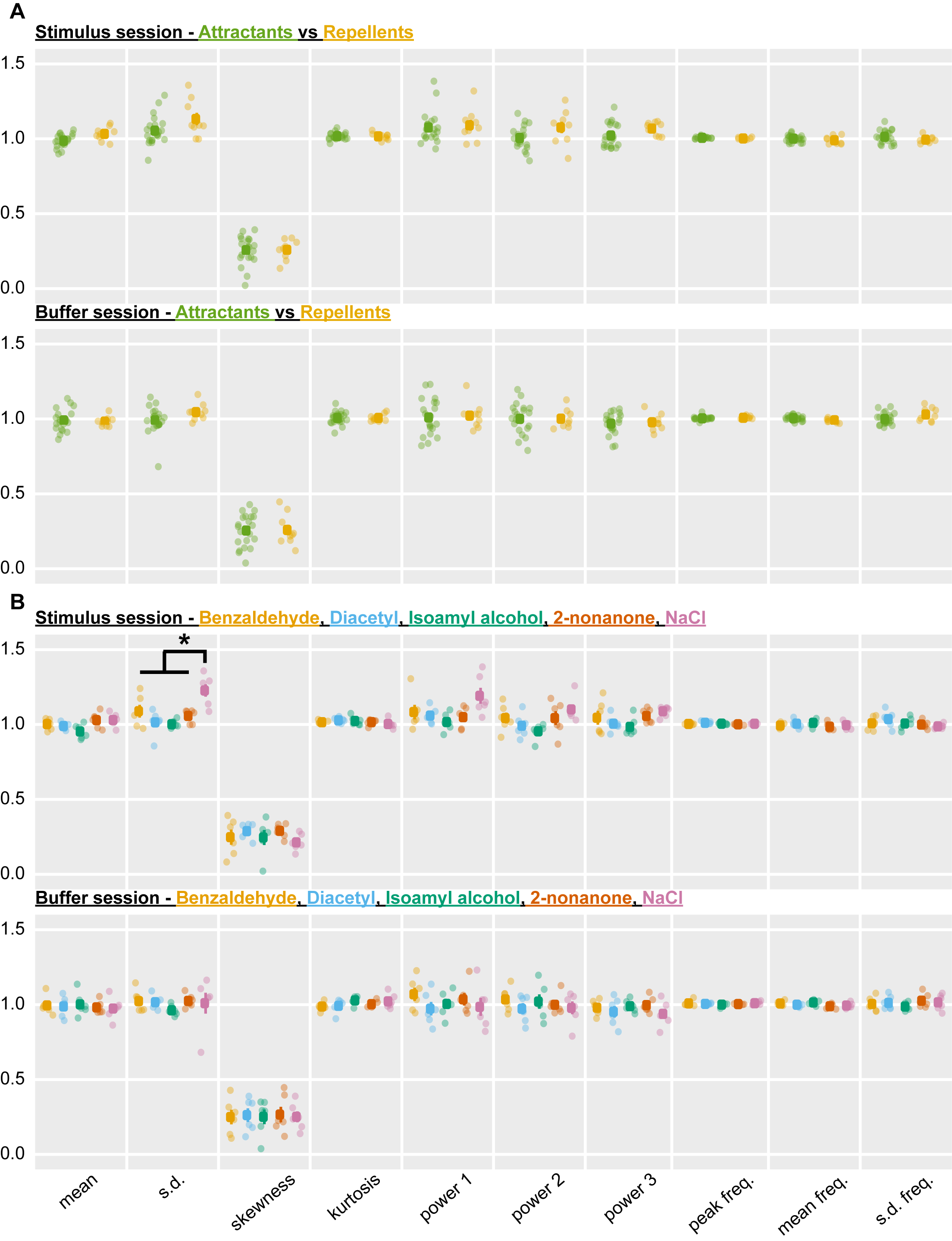

Supplement: S2 Fig — The first four features refer to mean, standard deviation, skewness, and kurtosis of neural activity. Power in 1st, 2nd, and 3rd bands refer to average power in the frequency ranges from 0.07–0.2 Hz, 0.2–0.34 Hz, and 0.34–0.47 Hz. Peak frequency is the frequency with the most power in a 30-second bin, and avg frequency and s.d. frequency are the average and standard deviation, respectively, of the frequencies with the most power in a sliding-window bin covering a 30-second period. Each color dot is the mean value across all seven pulses for a single worm, and the dark squares and lines indicate the mean and standard error of the mean across all worms. N = 21 for attractants and N = 9 for repellents (A), and N = 6 for each chemical stimulus (B). * p < 0.05 indicates features that were significant 1) by likelihood ratio test (LRT) on full and null generalized linear-mixed effects models (GLMEs), where the former included stimulus valence or identity as a fixed effect, and 2) by pairwise F-tests on stimulus coefficients of full GLME. Multiple comparisons correction for LRT used alpha = 0.05/meff, while those for F-tests used Bonferonni correction based on total number of distinct pairwise comparisons (i.e., alpha = 0.05/10). (TIF) [file pcbi.1009591.s002.tif]

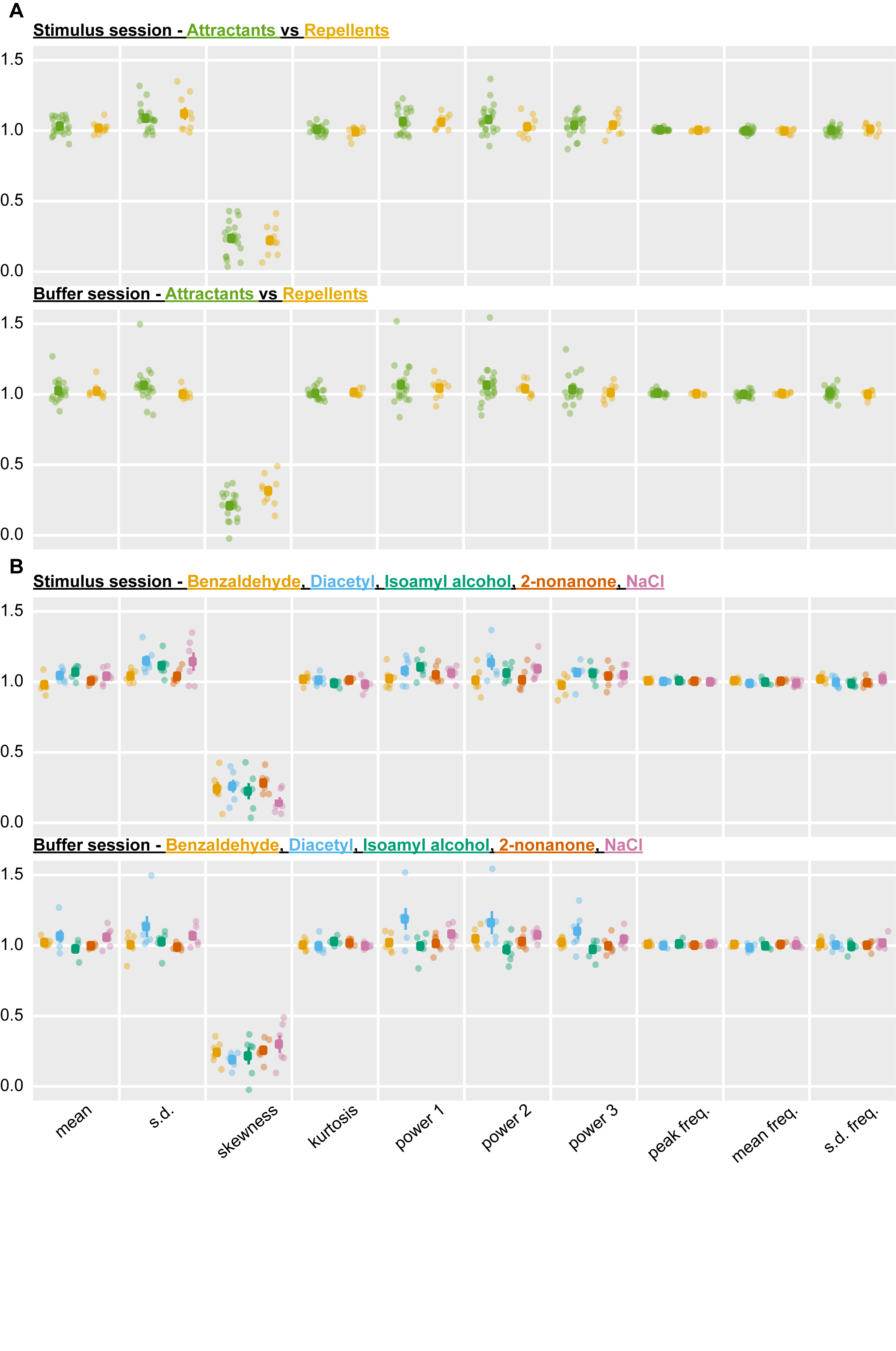

Supplement: S3 Fig — The first four features refer to mean, standard deviation, skewness, and kurtosis of neural activity. Power in 1st, 2nd, and 3rd bands refer to average power in the frequency ranges from 0.07–0.2 Hz, 0.2–0.34 Hz, and 0.34–0.47 Hz. Peak frequency is the frequency with the most power in a 30-second bin, and avg frequency and s.d. frequency are the average and standard deviation, respectively, of the frequencies with the most power in a sliding-window bin covering a 30-second period. Each color dot is the mean value across all seven pulses for a single worm, and the dark squares and lines indicate the mean and standard error of the mean across all worms. N = 21 for attractants and N = 9 for repellents (A), and N = 6 for each chemical stimulus (B). p > 0.05 for all features by likelihood ratio test (LRT) on full and null generalized linear-mixed effects models, where the former included stimulus valence or identity as a fixed effect; as a result, no F-tests were used. Multiple comparisons correction for LRT used alpha = 0.05/meff. (TIF) [file pcbi.1009591.s003.tif]

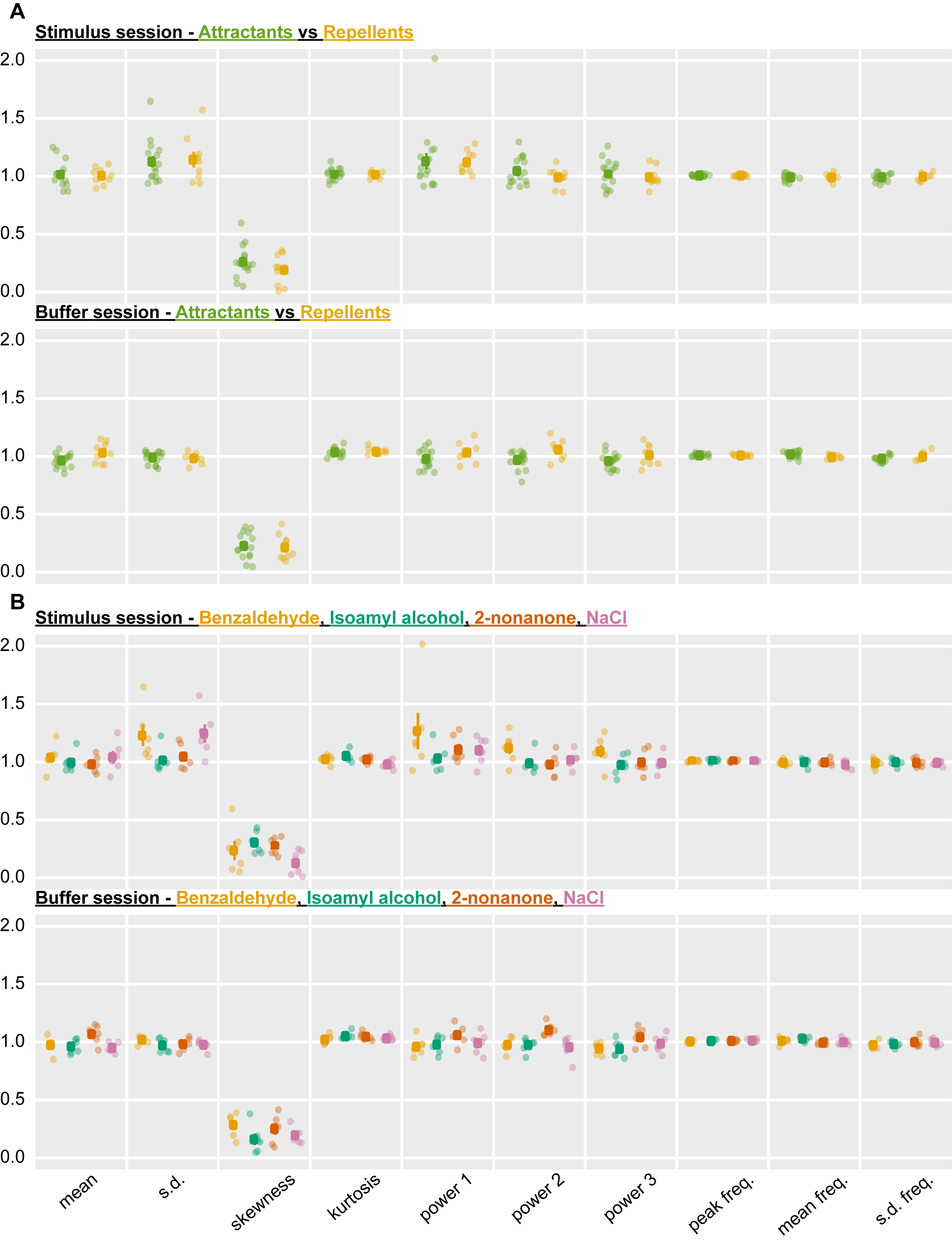

Supplement: S4 Fig — The one significant feature in Data Set 1 is not significant in Data Set 2. The first four features refer to mean, standard deviation, skewness, and kurtosis of neural activity. Power in 1st, 2nd, and 3rd bands refer to average power in the frequency ranges from 0.07–0.2 Hz, 0.2–0.34 Hz, and 0.34–0.47 Hz. Peak frequency is the frequency with the most power in a 30-second bin, and avg frequency and s.d. frequency are the average and standard deviation, respectively, of the frequencies with the most power in a sliding-window bin covering a 30-second period. Each color dot is the mean value across all seven pulses for a single worm, and the dark squares and lines indicate the mean and standard error of the mean across all worms. N = 15 for attractants and N = 9 for repellents (A), and N = 6 for each chemical stimulus (B). p > 0.05 for all features by likelihood ratio test (LRT) on full and null generalized linear-mixed effects models, where the former included stimulus valence or identity as a fixed effect; as a result, no F-tests were used. Multiple comparisons correction for LRT used alpha = 0.05/meff. (TIF) [file pcbi.1009591.s004.tif]

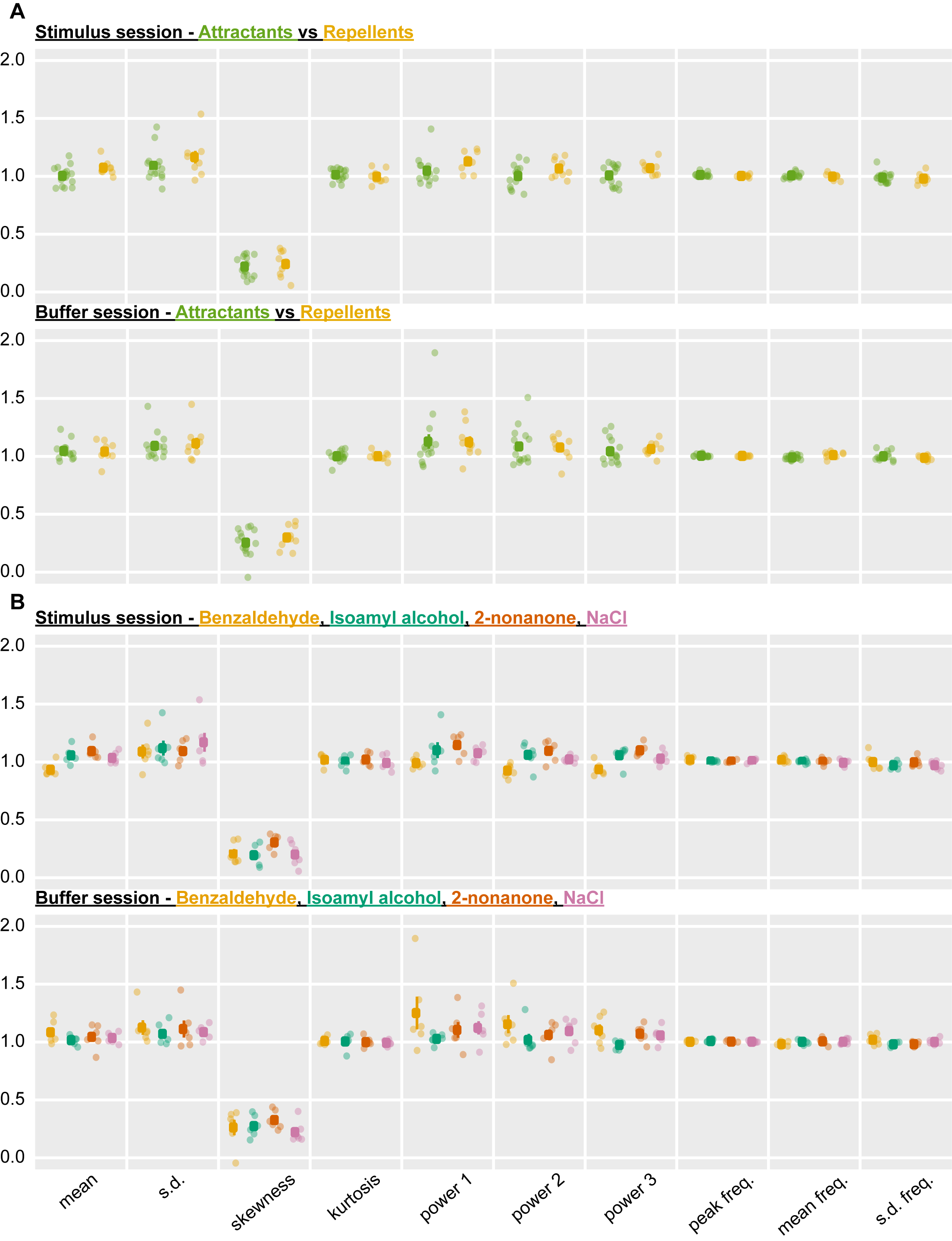

Supplement: S5 Fig — The first four features refer to mean, standard deviation, skewness, and kurtosis of neural activity. Power in 1st, 2nd, and 3rd bands refer to average power in the frequency ranges from 0.07–0.2 Hz, 0.2–0.34 Hz, and 0.34–0.47 Hz. Peak frequency is the frequency with the most power in a 30-second bin, and avg frequency and s.d. frequency are the average and standard deviation, respectively, of the frequencies with the most power in a sliding-window bin covering a 30-second period. Each color dot is the mean value across all seven pulses for a single worm, and the dark squares and lines indicate the mean and standard error of the mean across all worms. N = 15 for attractants and N = 9 for repellents (A), and N = 6 for each chemical stimulus (B). p > 0.05 for all features by likelihood ratio test (LRT) on full and null generalized linear-mixed effects models, where the former included stimulus valence or identity as a fixed effect; as a result, no F-tests were used. Multiple comparisons correction for LRT used alpha = 0.05/meff. (TIF) [file pcbi.1009591.s005.tif]

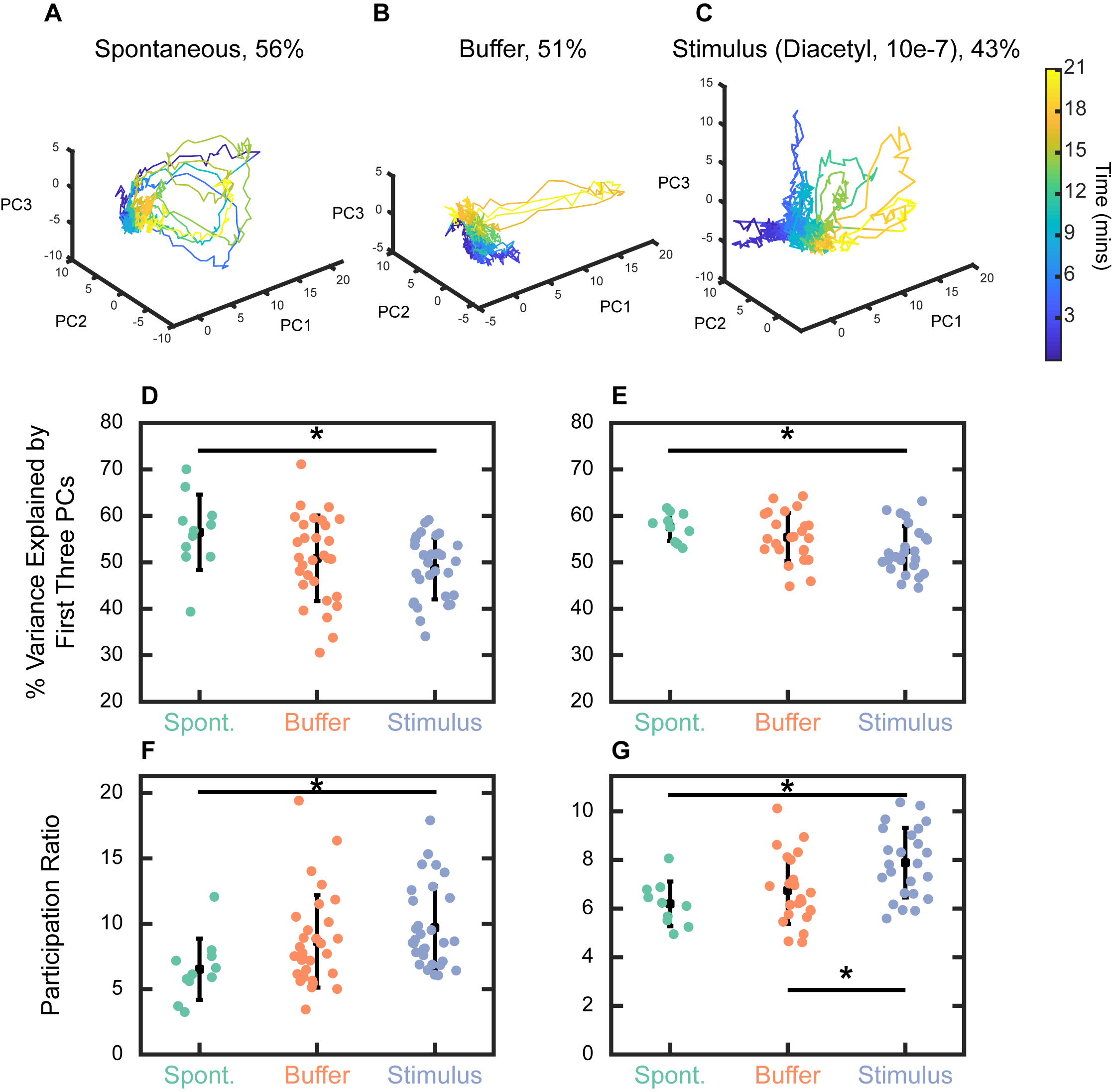

Supplement: S6 Fig — Example neural dynamics observed during a Spontaneous session forms loops through principal components analysis space (A), but not during a Buffer (B) or Stimulus session (C). The percentages in panels A to C refer to percent of total variance explained by first three principal components (PCs). Generally, the first three PCs explain a larger percentage of the variance during Spontaneous sessions than during Stimulus sessions in both Data Set 1 (D) and 2 (E). The Stimulus sessions also have a larger participation ratio than the Spontaneous and Buffer sessions in Data Set 1 (F) and 2 (G). Kruskal-Wallis test, with Dunn-Sidak post-hoc test, * p < 0.05 (specifically, p = 0.04 and 0.01 for D and E, p = 0.0046 for F, and p = 0.0059 for Spont. vs Stimulus, and 0.03 for Buffer vs Stimulus in G). (TIF) [file pcbi.1009591.s006.tif]

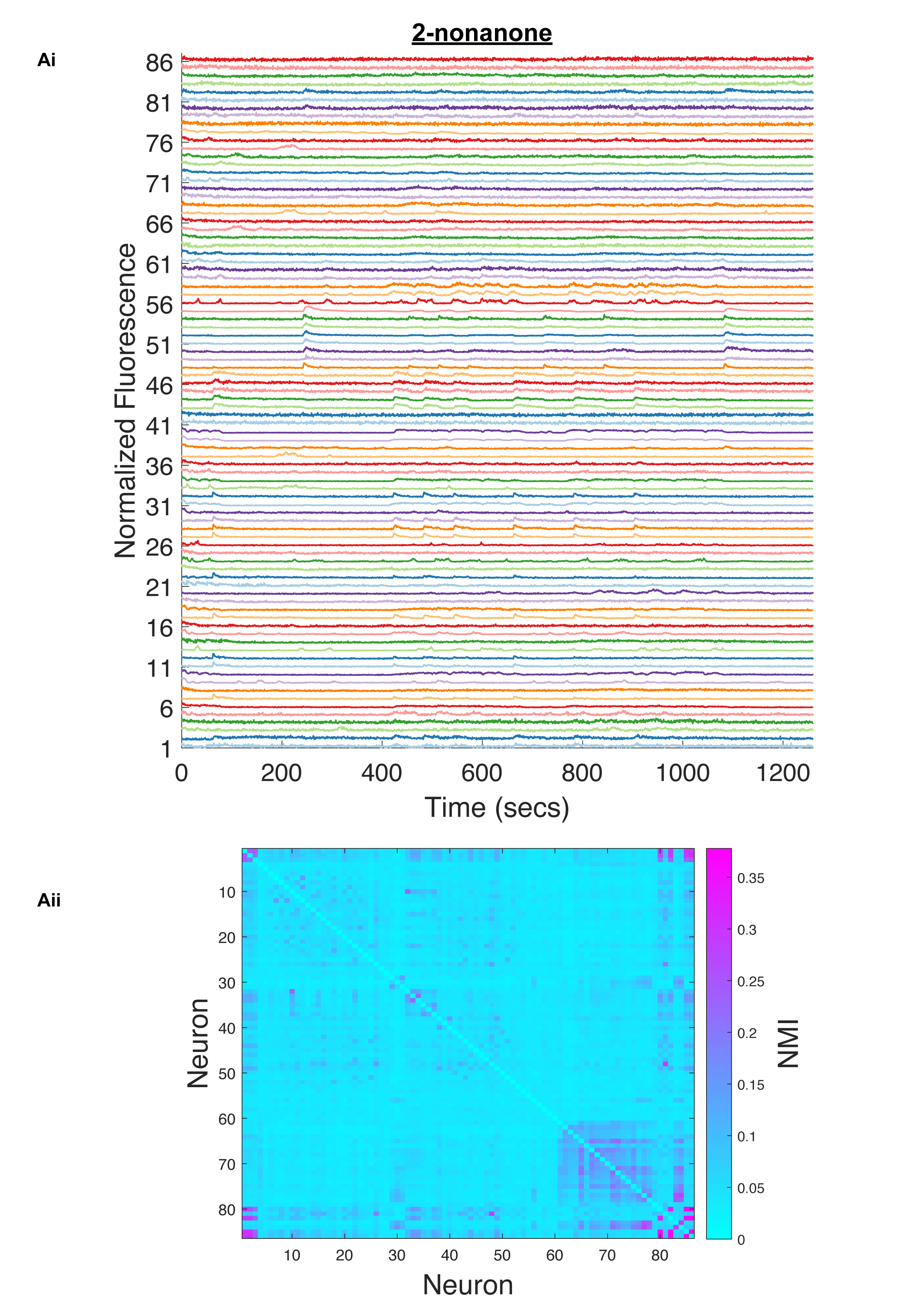

Supplement: S7 Fig — Ai, Bi, Ci, Di, and Ei depict all neurons recorded in five worms exposed to 2-nonanone (A), benzaldehyde (B), diacetyl (C), salt (D), or isoamyl alcohol (E). The time series were all normalized to have a maximum value of 1 by dividing each time series by its own maximum in the 21-minute long imaging session. Aii, Bii, Cii, Dii, and Eii depict the adjacency matrices computed from the time series by taking the normalized mutual information (NMI) between each pair of neurons; thus, for n neural time series, there is a corresponding nxn matrix of NMI values, with a minimum of 0 and maximum of 1. Note that in these examples we used the entire time series to compute the adjacency matrices, but in our analyses we used 30-second long periods of time. (TIFF) [file pcbi.1009591.s007.tiff]

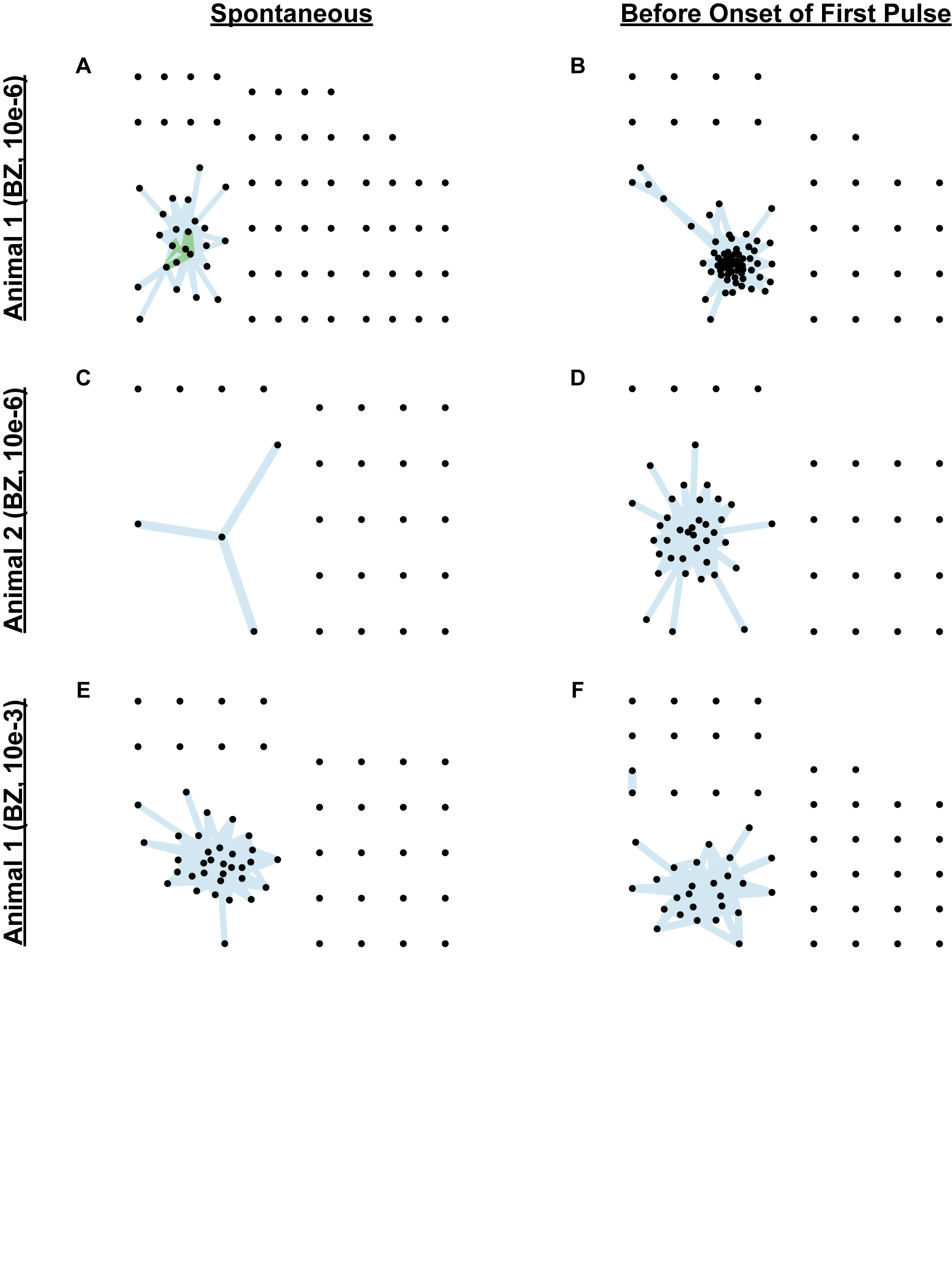

Supplement: S8 Fig — The networks depicted here, for three example worms, show neurons (circles) connected by lines (edges), where the lines represent the normalized mutual information (NMI) between their time series. Only the strongest (i.e., NMI > = 0.4) interactions in the network are shown (hence, why many edges are missing), with those in green indicating the presence of a triangle (i.e., a triplet of connected neurons whose total weight > = 1.8) and those in blue its absence. All networks are based on neural activity observed in the 30-second period between 30 seconds and 1 minute into the beginning of either the Buffer (A, C, E) or Stimulus (B, D, F) session. Worms in panels A, C, and E were about to experience M9 buffer. Worms in panels B and D were about to experience benzaldehyde (BZ) at a concentration of 10e-6, while the worm in panel F was about to experience BZ at a concentration of 10–3. Some worms have more triangles prior to buffer onset (A) than stimulus onset (B), others have different numbers of connections that survive the 0.4 threshold (C, D), and others look similar (E, F). (TIF) [file pcbi.1009591.s008.tif]

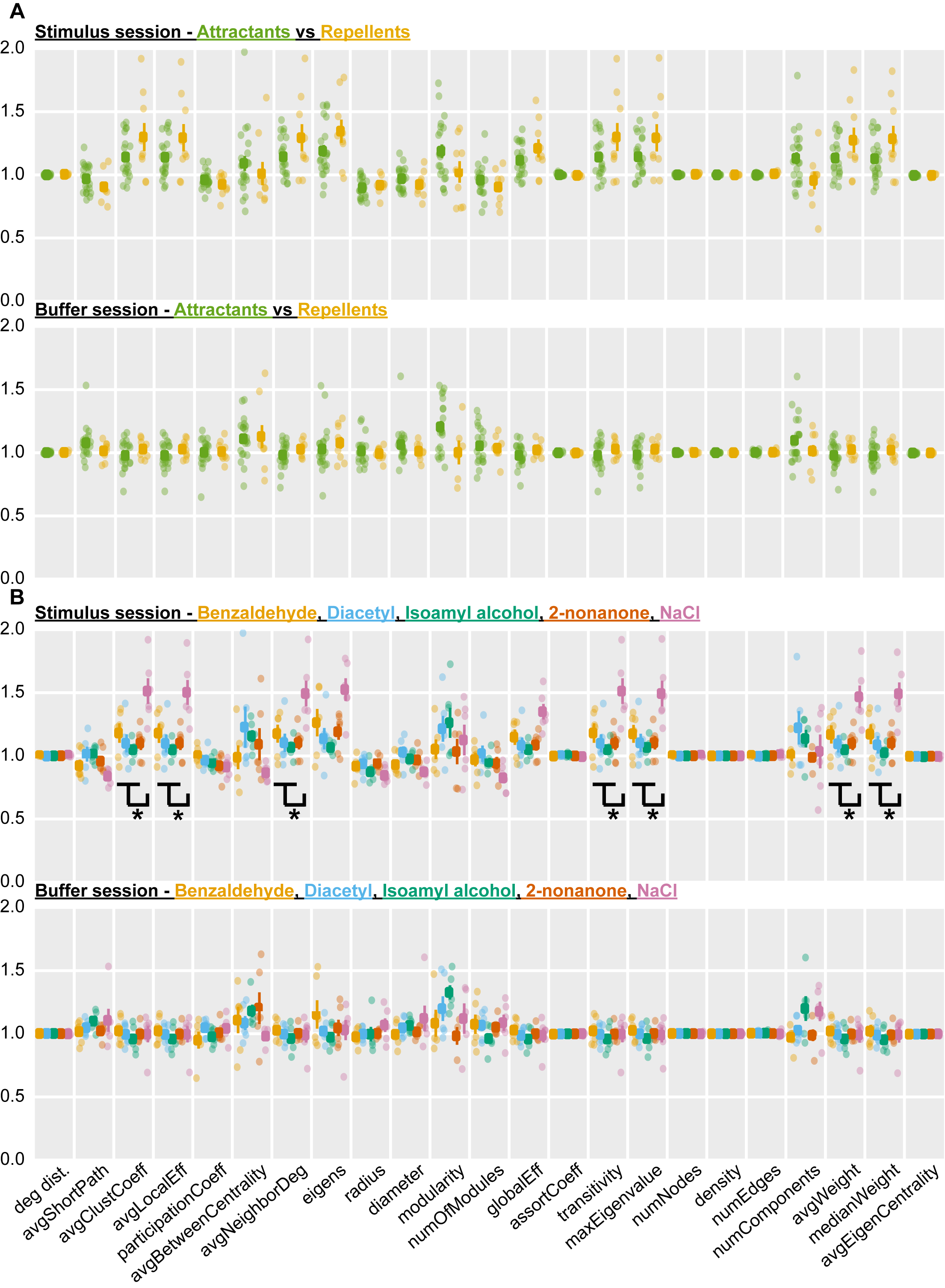

Supplement: S9 Fig — Network features are derived from adjacency matrices constructed with normalized mutual information. See S1 Table for description of features. Each color dot is the mean value across all seven pulses for a single worm, and the dark squares and lines indicate the mean and standard error of the mean across all worms. N = 21 for attractants and N = 9 for repellents (A), and N = 6 for each chemical stimulus (B). * p < 0.05 indicates features that were significant 1) by likelihood ratio test (LRT) on full and null generalized linear-mixed effects models (GLMEs), where the former included stimulus valence or identity as a fixed effect, and 2) by pairwise F-tests on stimulus coefficients of full GLME. Multiple comparisons correction for LRT used alpha = 0.05/meff, while those for F-tests used Bonferonni correction based on total number of distinct pairwise comparisons (i.e., alpha = 0.05/10). (TIF) [file pcbi.1009591.s009.tif]

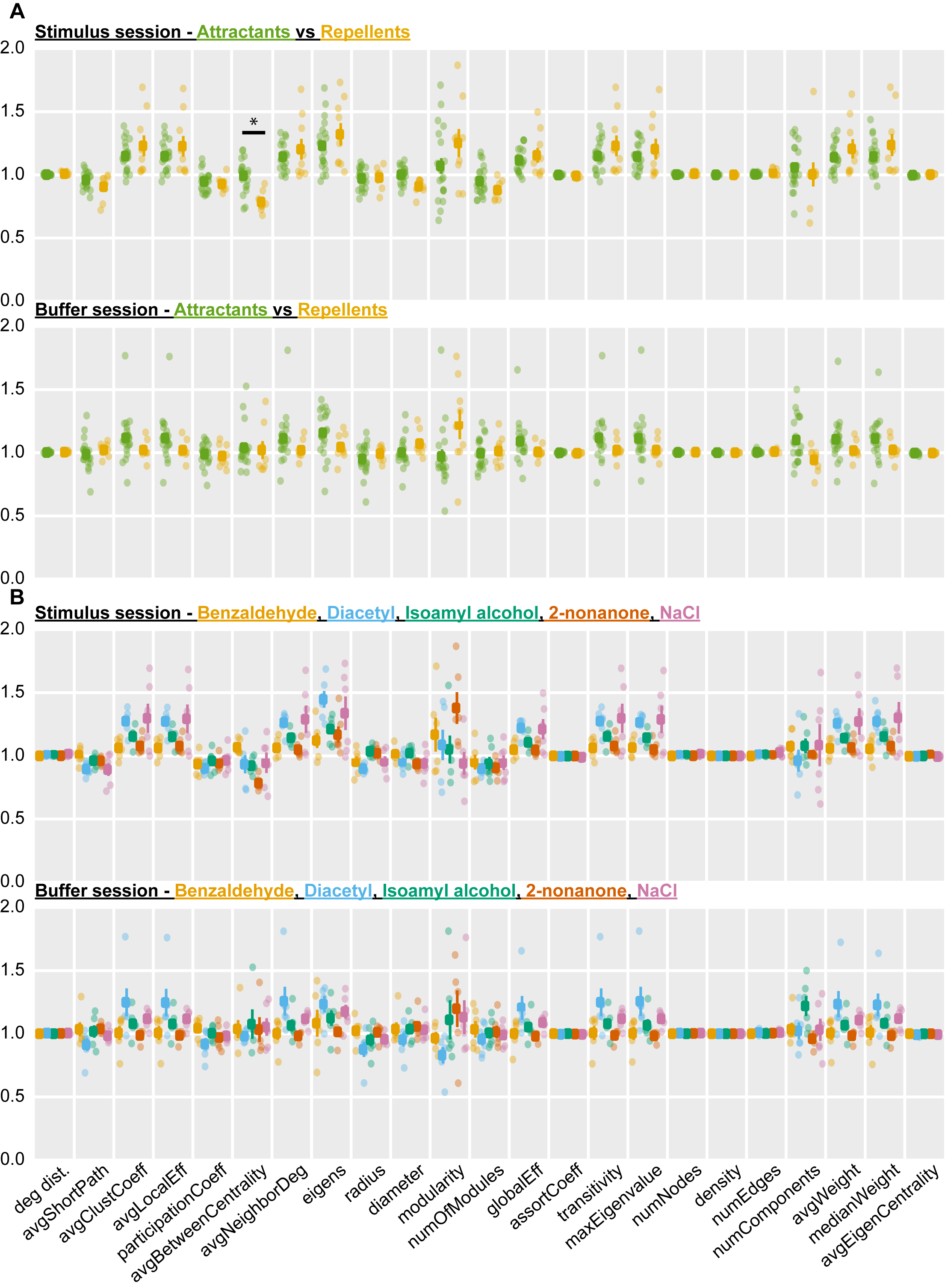

Supplement: S10 Fig — Network features are derived from adjacency matrices constructed with normalized mutual information. See S1 Table for description of features. Each color dot is the mean value across all seven pulses for a single worm, and the dark squares and lines indicate the mean and standard error of the mean across all worms. N = 21 for attractants and N = 9 for repellents (A), and N = 6 for each chemical stimulus (B). p > 0.05 for all features by likelihood ratio test (LRT) on full and null generalized linear-mixed effects models, where the former included stimulus valence or identity as a fixed effect; as a result, no F-tests were used. Multiple comparisons correction for LRT used alpha = 0.05/meff. (TIF) [file pcbi.1009591.s010.tif]

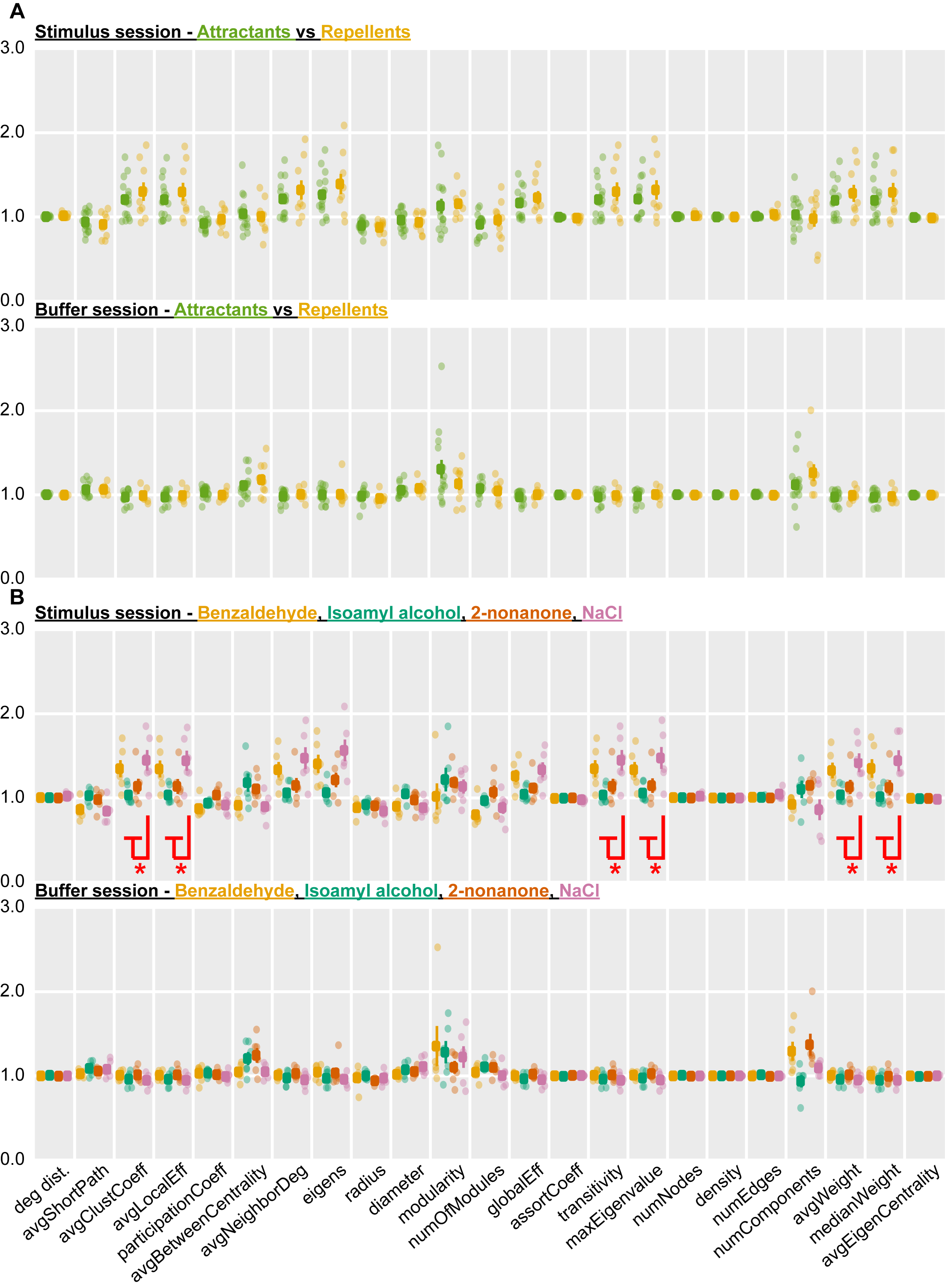

Supplement: S11 Fig — Six of the seven significant features from Data Set 1 are also significant in Data Set 2. Network features are derived from adjacency matrices constructed with normalized mutual information. See S1 Table for description of features. Each color dot is the mean value across all seven pulses for a single worm, and the dark squares and lines indicate the mean and standard error of the mean across all worms. N = 15 for attractants and N = 9 for repellents (A), and N = 6 for each chemical stimulus (B). * p < 0.05 indicates features that were significant 1) by likelihood ratio test (LRT) on full and null generalized linear-mixed effects models (GLMEs), where the former included stimulus valence or identity as a fixed effect, and 2) by pairwise F-tests on stimulus coefficients of full GLME. Multiple comparisons correction for LRT used alpha = 0.05/meff, while those for F-tests used Bonferonni correction based on total number of distinct pairwise comparisons (i.e., alpha = 0.05/3). (TIF) [file pcbi.1009591.s011.tif]

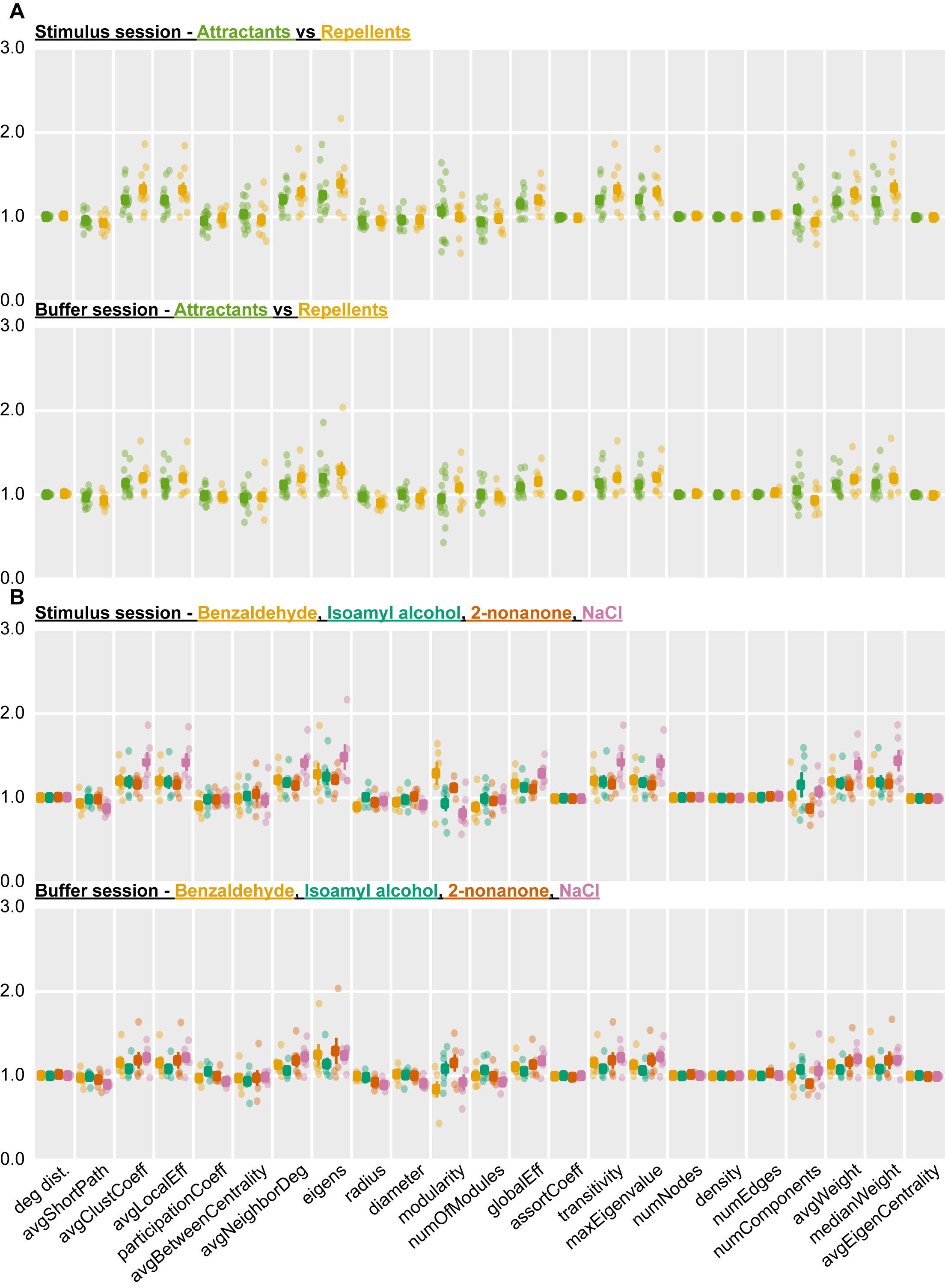

Supplement: S12 Fig — Network features are derived from adjacency matrices constructed with normalized mutual information. See S1 Table for description of features. Each color dot is the mean value across all seven pulses for a single worm, and the dark squares and lines indicate the mean and standard error of the mean across all worms. N = 15 for attractants and N = 9 for repellents (A), and N = 6 for each chemical stimulus (B). p > 0.05 for all features by likelihood ratio test (LRT) on full and null generalized linear-mixed effects models, where the former included stimulus valence or identity as a fixed effect; as a result, no F-tests were used. Multiple comparisons correction for LRT used alpha = 0.05/meff. (TIF) [file pcbi.1009591.s012.tif]

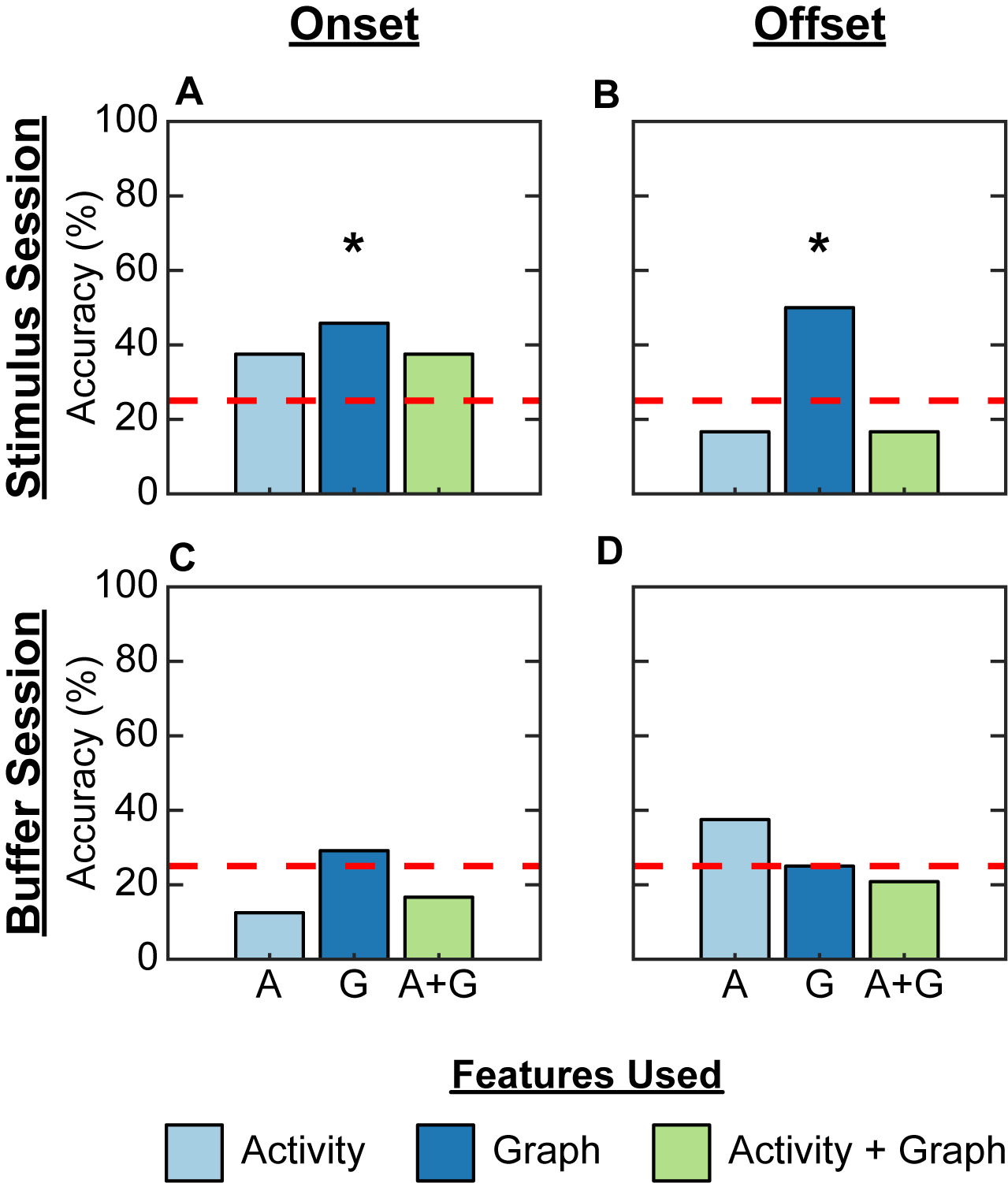

Supplement: S13 Fig — A Logistic Regression classifier was trained to identify chemical stimulus using nested leave-one-out cross-validation on Data Set 1 with all six diacetyl sessions removed for stimulus onset (A, C) and offset (B, D). We used Activity features (light blue bars), Graph features (dark blue bars), and both Activity and Graph features together (green bars). The classifier achieved statistically significant above-chance accuracy on stimulus onset and offset with Graph features alone (p = 0.01 and 0.03 for panels A and B), which was higher than in the full Data Set 1. The dashed red line refers to chance accuracy, which is 25% because Data Set 1 was trimmed to the 4 chemical stimuli studied in Data Set 2 (i.e., benzaldehyde, isoamyl alcohol, 2-nonanone, and NaCl). Statistical significance was assessed using a permutation test with n = 100 permutations. N = 24 worms. * refer to classifier with accuracy significantly above chance according to permutation test. (TIF) [file pcbi.1009591.s013.tif]

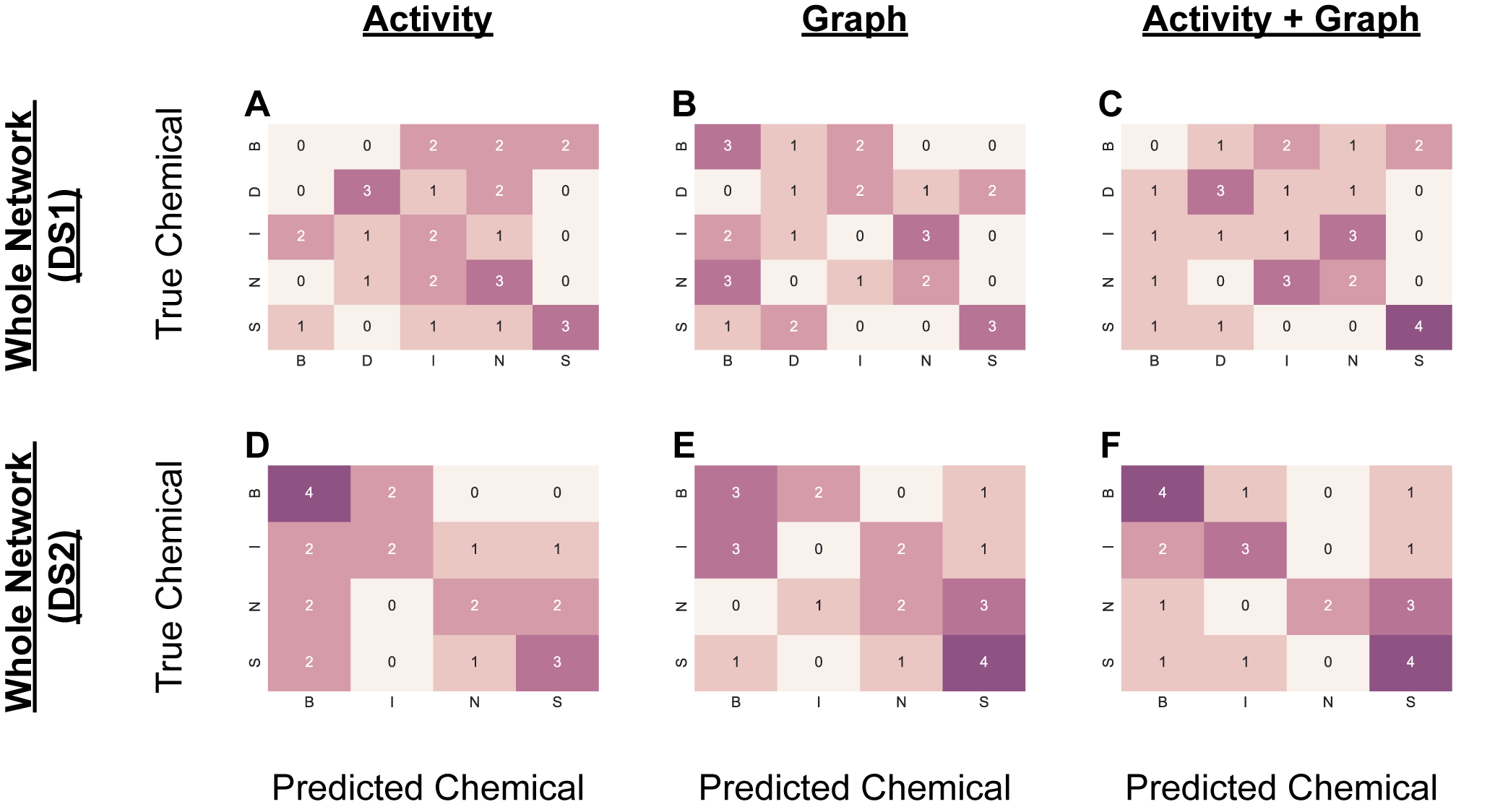

Supplement: S14 Fig — We trained a Logistic Regression classifier with nested leave-one-out cross-validation based on either activity (A, D), graph (B, E), or combined (C, F) features for the Whole Network on Data Sets 1 (DS1; A—C) and 2 (DS2; D—F). The true chemicals are labeled on the rows, and the predicted chemicals on the columns. Chemicals along the main diagonal are correct predictions. The chemicals are benzaldehyde (B), diacetyl (D), isoamyl alcohol (I), 2-nona- none (N), and NaCl (S, for salt). Since there are six animals per condition, the largest possible value is 6, with darker squares indicating more predictions of that column’s label. Note that B and S are often classified best (i.e., have darker squares on the main diagonal) using graph features. All features were standardized. N = 30 worms for Data Set 1, 24 worms for Data Set 2, and 6 worms per chemical. (TIF) [file pcbi.1009591.s014.tif]
