# Supplementary material for: Neural network features distinguish chemosensory stimuli in Caenorhabditis elegans
Source: PLoS Comput Biol. 2021 Nov 9;17(11):e1009591. doi: 10.1371/journal.pcbi.1009591 (PMC8604368; doi:10.1371/journal.pcbi.1009591)
Supplement: S1 Table — The twenty-two listed features are grouped into one of five classes: basic structure (of the adjacency matrix), functional segregation (measures of the decomposability of the network), functional integration (the potential for disparate parts of the network to communicate), centrality (the importance of any one neuron to network communication), and resilience to perturbations, such as lesions (measures of how robust the system is to disruptions at individual nodes). For a review, see (1). (DOCX) [file pcbi.1009591.s015.docx]

| **Graph Theory Feature** | **Description** |
| --- | --- |
| *Basic structure* | |
| numNodes | the number of neurons that had at least one non-zero weight to another neuron |
| numEdges | the number of edges |
| density | the density of edges (i.e., the number of actual edges divided by the total possible number of edges in a fully-connected network) |
| numComponents | the number of isolated subgraphs in the network |
| avgWeight | the average weight of the network |
| medWeight | the median weight of the network |
| avgEigenvalue | the mean of the positive eigenvalues of the adjacency matrix |
| maxEigenvalue | the largest eigenvalue of the adjacency matrix |
| *Functional segregation* | |
| avgClusteringCoeff | the average over all neurons of the fraction of 3-neuron clusters, or triangles, around each neuron |
| transitivity | a globally normalized version of the clustering coefficient |
| avgLocalEff | the mean local efficiency (i.e., the average over all neurons of the lengths of the inverse shortest paths between two of the neuron’s neighbors) |
| modularity | the extent to which a network can be divided into clusters, or modules, of neurons with dense connections amongst themselves, and sparse connections to neurons in other clusters |
| numModules | the number of modules |
| *Functional integration* | |
| avgShortestPath | the average shortest path between all pairs of neurons in the network |
| globalEff | the global efficiency, or the average inverse shortest path length |
| radius | the smallest shortest path connecting any two neurons |
| diameter | the largest shortest path connecting any two neurons |
| *Centrality* | |
| avgParticipationCoeff | the degree to which a neuron communicates with neurons in different modules |
| avgBetweennessCentrality | the average of the fraction of shortest paths linking any two neurons that pass through a given neuron |
| avgEigenvectorCentrality | the average of each node’s importance as defined by its connection to other important nodes |
| *Resilience to perturbations* | |
| avgDegDist | the average of each neuron's degree, or sum of the edge weights to other neurons |
| assortCoeff | the correlation coefficient between the degrees of two connected neurons, where a negative number indicates neurons with a high degree are connected to neurons with a low degree |
| avgNeighborDegree | the average degree of the neighbors of a given node, averaged over all nodes |
