# Supplementary material for: Neural network features distinguish chemosensory stimuli in Caenorhabditis elegans
Source: PLoS Comput Biol. 2021 Nov 9;17(11):e1009591. doi: 10.1371/journal.pcbi.1009591 (PMC8604368; doi:10.1371/journal.pcbi.1009591)
Supplement: S4 Table — Only the top 30%, 20%, or 10% of weights were kept in each network (columns). Results from the likelihood ratio test applied on a full vs null model. The null model includes information on animal ID and time since first pulse. The full model includes information on either Valence or Identity in addition to the null model. The p-values in red indicate a significant difference in the data’s likelihood when explained with the full model vs the null model; hence, the parameter (i.e., Valence or Identity) significantly improved model fit. DS1 and DS2 refers to Data Sets 1 and 2. The p-values in bold red indicate a significant difference in DS1 and DS2. Any missing entries did not converge. The meff for Identity comparison on Stimulus Onset and Stimulus Offset were, for 30%, 26.3, 27.2; for 20%, 26.4, 27.4; for 10%, 26.7, 27.3. (DOCX) [file pcbi.1009591.s018.docx]

| Comparison | Pulse Switch | Graph Theory Feature | 30% | 20% | 10% |
| --- | --- | --- | --- | --- | --- |
| Valence | Onset | assortCoeff | 0.0898 | 0.1831 | 0.8319 |
|  |  | avgBetweenCentrality | 0.2051 |  | 0.0913 |
|  |  | avgClusteringCoeff | 0.1201 | 0.1619 | 0.3365 |
|  |  | avgDegDist | 0.1268 | 0.0973 | 0.2789 |
|  |  | avgEigenvalue | 0.1078 | 0.1844 | 0.1896 |
|  |  | avgEigenvectorCentrality | 0.2329 | 0.0859 | 0.2750 |
|  |  | avgLocalEff | 0.1178 | 0.1602 | 0.3547 |
|  |  | avgNeighborDeg | 0.2172 | 0.3197 | 0.6413 |
|  |  | avgParticipationCoeff | 0.3008 | 0.4763 | 0.8380 |
|  |  | avgShortestPaths | 0.0869 | 0.0675 | 0.2384 |
|  |  | avgWeight | 0.1203 | 0.1675 | 0.3658 |
|  |  | density | 0.1364 | 0.1287 | 0.3320 |
|  |  | diameter | 0.4409 | 0.2425 | 0.6530 |
|  |  | globalEff | 0.1204 | 0.1320 | 0.2551 |
|  |  | maxEigenvalue | 0.1269 | 0.1565 | 0.2119 |
|  |  | medianWeight |  |  |  |
|  |  | modularity | 0.1703 | 0.2066 | 0.1246 |
|  |  | numComponents | 0.9731 | 0.5439 | 0.4381 |
|  |  | numEdges | 0.9745 | 0.6293 | 0.5289 |
|  |  | numModules | 0.6673 | 0.4152 | 0.3820 |
|  |  | numNodes | 0.1192 | 0.0623 | 0.2259 |
|  |  | radius | 0.4842 | 0.4842 | 0.4053 |
|  |  | transitivity | 0.0983 | 0.1361 | 0.2037 |
|  | Offset | assortCoeff | 0.3468 | 0.4611 | 0.0581 |
|  |  | avgBetweenCentrality | 0.9661 | 0.4291 | 0.5151 |
|  |  | avgClusteringCoeff | 0.8705 | 0.5393 | 0.4288 |
|  |  | avgDegDist | 0.0708 | 0.0526 | 0.1745 |
|  |  | avgEigenvalue |  | 0.7173 | 0.9051 |
|  |  | avgEigenvectorCentrality | 0.3182 | 0.1208 | 0.2459 |
|  |  | avgLocalEff | 0.9136 | 0.7312 | 0.5011 |
|  |  | avgNeighborDeg | 0.7750 | 0.6075 | 0.3010 |
|  |  | avgParticipationCoeff | 0.9133 | 0.1482 | 0.1670 |
|  |  | avgShortestPaths | 0.7611 | 0.8331 | 0.8187 |
|  |  | avgWeight | 0.8202 | 0.5499 | 0.3711 |
|  |  | density | 0.0972 | 0.0753 | 0.1566 |
|  |  | diameter | 0.4881 | 0.6200 |  |
|  |  | globalEff | 0.8881 | 0.8934 | 0.6540 |
|  |  | maxEigenvalue | 0.9835 | 0.8102 | 0.5916 |
|  |  | medianWeight |  |  |  |
|  |  | modularity | 0.0228 | 0.0716 | 0.1832 |
|  |  | numComponents | 0.2524 | 0.0077 | 0.1837 |
|  |  | numEdges | 0.6084 | 0.3940 | 0.4972 |
|  |  | numModules | 0.9201 | 0.3082 | 0.4862 |
|  |  | numNodes | 0.0374 | 0.0271 | 0.2636 |
|  |  | radius | 0.8341 | 0.9592 | 0.9543 |
|  |  | transitivity | 0.8660 | 0.6794 | 0.5814 |
| Identity | Onset | assortCoeff | 0.0188 | 0.2646 | 0.9864 |
|  |  | avgBetweenCentrality | 0.2028 | 0.9017 | 0.1285 |
|  |  | avgClusteringCoeff | 0.0009 | 0.0014 | 0.0144 |
|  |  | avgDegDist | 0.0407 | 0.0622 | 0.1355 |
|  |  | avgEigenvalue | 0.0019 | 0.0163 | 0.0208 |
|  |  | avgEigenvectorCentrality | 0.1243 | 0.0780 | 0.1580 |
|  |  | avgLocalEff | 0.0014 | 0.0022 | 0.0126 |
|  |  | avgNeighborDeg | 0.0028 | 0.0093 | 0.1049 |
|  |  | avgParticipationCoeff | 0.0036 | 0.0122 | 0.0069 |
|  |  | avgShortestPaths | 0.0130 | 0.0163 | 0.0789 |
|  |  | avgWeight | 0.0028 | 0.0107 | 0.0298 |
|  |  | density | 0.0473 | 0.0868 | 0.1508 |
|  |  | diameter | 0.2812 | 0.0933 | 0.3952 |
|  |  | globalEff | 0.0026 | 0.0052 | 0.0126 |
|  |  | maxEigenvalue | 0.0014 | 0.0025 | 0.0062 |
|  |  | medianWeight |  |  |  |
|  |  | modularity | 0.5352 | 0.5872 | 0.3843 |
|  |  | numComponents | 0.1457 | 0.0186 | 0.0101 |
|  |  | numEdges | 0.8592 | 0.9968 | 0.4166 |
|  |  | numModules | 0.0231 | 0.1507 | 0.0018 |
|  |  | numNodes | 0.0329 | 0.0374 | 0.1219 |
|  |  | radius | 0.0824 | 0.0824 | 0.0306 |
|  |  | transitivity | 0.0009 | 0.0019 | 0.0019 |
|  | Offset | assortCoeff | 0.0337 | 0.1013 | 0.2419 |
|  |  | avgBetweenCentrality | 0.0354 | 0.3777 | 0.7891 |
|  |  | avgClusteringCoeff | 0.0002 | 0.0001 | 0.0010 |
|  |  | avgDegDist | 0.0069 | 0.0009 | 0.0031 |
|  |  | avgEigenvalue | 0.0435 | 0.0085 | 0.0053 |
|  |  | avgEigenvectorCentrality | 0.1405 | 0.0083 | 0.0038 |
|  |  | avgLocalEff | 0.0004 | 0.0003 | 0.0011 |
|  |  | avgNeighborDeg | 0.0007 | 0.0025 | 0.0089 |
|  |  | avgParticipationCoeff | 0.9178 | 0.9243 | 0.1653 |
|  |  | avgShortestPaths | 0.0279 | 0.0070 | 0.0096 |
|  |  | avgWeight | 0.0004 | 0.0002 | 0.0005 |
|  |  | density | 0.0041 | 0.0006 | 0.0011 |
|  |  | diameter | 0.0265 | 0.0273 | 0.0663 |
|  |  | globalEff | 0.0008 | 0.0003 | 0.0005 |
|  |  | maxEigenvalue | 0.0005 | 0.0004 | 0.0006 |
|  |  | medianWeight |  |  |  |
|  |  | modularity | 0.0279 | 0.1865 | 0.5009 |
|  |  | numComponents | 0.0003 | 0.0001 | 0.0002 |
|  |  | numEdges | 0.8848 | 0.5229 | 0.2857 |
|  |  | numModules | 0.8569 | 0.9371 | 0.7847 |
|  |  | numNodes | 0.0171 | 0.0021 | 0.0285 |
|  |  | radius | 0.0155 | 0.0167 | 0.0160 |
|  |  | transitivity | 0.0002 | 0.0002 | 0.0014 |
