# Supplementary material for: Neural network features distinguish chemosensory stimuli in Caenorhabditis elegans
Source: PLoS Comput Biol. 2021 Nov 9;17(11):e1009591. doi: 10.1371/journal.pcbi.1009591 (PMC8604368; doi:10.1371/journal.pcbi.1009591)
Supplement: S14 Table — Best performance achieved by logistic regression classifier on a specific classification task–namely, for a given session, pulse switch type, and one of three sets of features, correctly classify responses. The nested leave-one-out cross validation accuracy, the mean and standard deviation of the accuracies of a null distribution built using 100 permutations of the labels, and the corresponding p-value, or relative position of its accuracy in the null distribution, are all listed. Values in red attained significantly above-chance accuracies, and those in bold red did so in Data Sets 1 and 2. Some tasks did not exceed chance (e.g., stimulus onset during Buffer sessions on Data Set 1), and this is indicated by a dashed line to indicate that no permutation testing was conducted. Accuracy on some classification tasks was higher when features were standardized (y = yes, n = no, y/n = same accuracy with or without standardization). Chance is 20% for Data Set 1 (DS1) and 25% for Data Set (DS2). GT = Graph Theory, Comb = Activity + Graph Theory. (DOCX) [file pcbi.1009591.s028.docx]

| Session | Pulse Switch | Data Set | Features | Best Possible Accuracy (%) | Permutation score (%, mean±s.d.) | p-value | Standardized |
| --- | --- | --- | --- | --- | --- | --- | --- |
| Buffer | Onset | DS1 | GT | 17 | - | - | n |
|  |  |  | Activity | 17 | - | - | y |
|  |  |  | Comb. | 7 | - | - | y/n |
|  |  | DS2 | GT | 33 | 19±11 | 0.1485 | n |
|  |  |  | Activity | 25 | - | - | n |
|  |  |  | Comb. | 33 | 21±10 | 0.1881 | y |
|  | Offset | DS1 | GT | 13 | - | - | n |
|  |  |  | Activity | 30 | 18±8 | 0.1089 | y |
|  |  |  | Comb. | 7 | - | - | n |
|  |  | DS2 | GT | 13 | - | - | n |
|  |  |  | Activity | 17 | - | - | y |
|  |  |  | Comb. | 25 | - | - | y |
| Stimulus | Onset | DS1 | GT | 33 | 14±8 | 0.0297 | n |
|  |  |  | **Activity** | **37** | **17±9** | **0.0297** | **y** |
|  |  |  | Comb. | 40 | 13±8 | 0.0099 | n |
|  |  | DS2 | GT | 33 | 21±11 | 0.1782 | y |
|  |  |  | **Activity** | **46** | **19±10** | **0.0297** | **y** |
|  |  |  | Comb. | 25 | - | - | y |
|  | Offset | DS1 | GT | 23 | 16±9 | 0.2376 | n |
|  |  |  | Activity | 7 | - | - | y |
|  |  |  | Comb. | 17 | - | - | n |
|  |  | DS2 | GT | 13 | - | - | y/n |
|  |  |  | Activity | 38 | 19±9 | 0.0495 | n |
|  |  |  | Comb. | 13 | - | - | y/n |
