# Supplementary material for: Neural network features distinguish chemosensory stimuli in Caenorhabditis elegans
Source: PLoS Comput Biol. 2021 Nov 9;17(11):e1009591. doi: 10.1371/journal.pcbi.1009591 (PMC8604368; doi:10.1371/journal.pcbi.1009591)
Supplement: S16 Table — Performance achieved by logistic regression classifier on a specific classification task–namely, for a given session, pulse switch type, and one of three sets of features, correctly classify responses. The nested leave-one-out cross validation accuracy, the mean and standard deviation of the accuracies of a null distribution built using 100 permutations of the labels, and the corresponding p-value, or relative position of its accuracy in the null distribution, are all listed. We only used non-standardized graph features computed on the Simplified Network. Values in red attained significantly above-chance accuracies, and those in bold red did so in Data Sets 1 and 2. Some tasks did not exceed chance (e.g., stimulus onset during Buffer sessions on Data Set 1), and this is indicated by a dashed line to indicate that no permutation testing was conducted. Chance is 20% for Data Set 1 (DS1) and 25% for Data Set (DS2). (DOCX) [file pcbi.1009591.s030.docx]

| Session | Pulse Switch | Data Set | Accuracy (%) | Permutation score (%, mean±s.d.) | p-value |
| --- | --- | --- | --- | --- | --- |
| Buffer | Onset | DS1 | 10 | - | - |
|  |  | DS2 | 21 | - | - |
|  | Offset | DS1 | 7 | - | - |
|  |  | DS2 | 21 | - | - |
| Stimulus | Onset | **DS1** | **47** | **14±9** | **0.0099** |
|  |  | **DS2** | **54** | **20±11** | **0.0099** |
|  | Offset | DS1 | 13 | - | - |
|  |  | DS2 | 8 | - | - |
