# Supplementary material for: Neural network features distinguish chemosensory stimuli in Caenorhabditis elegans
Source: PLoS Comput Biol. 2021 Nov 9;17(11):e1009591. doi: 10.1371/journal.pcbi.1009591 (PMC8604368; doi:10.1371/journal.pcbi.1009591)
Supplement: S19 Table — Results from the likelihood ratio test applied on a full vs null model. The null model includes information on animal ID and time since first pulse. The full model includes information on Identity in addition to the null model. The p-values in red indicate a significant difference in the data’s likelihood when explained with the full model vs the null model; hence, the parameter (i.e., Identity) significantly improved model fit. DS1 and DS2 refers to Data Sets 1 and 2. The p-values in bold red indicate a significant difference in DS1 and DS2. All networks formed with time bin 30 seconds, as in the main text. Features shown are for stimulus onset. The meff for 0.05, 0.1, and 0.2 bins was 27.1, 27.4, 25.5 for DS1, and 6.2, 13.6, 9.9 for DS2. (DOCX) [file pcbi.1009591.s033.docx]

| Data Set | Feature | NMI 0.05 | NMI 0.1 | NMI 0.2 |
| --- | --- | --- | --- | --- |
| DS1 | avgWeight | **0.0005** | **0.0010** | 0.0139 |
|  | avgClusteringCoeff | **0.0004** | **0.0005** | 0.0108 |
|  | avgLocalEff | **0.0004** | **0.0006** | 0.0117 |
|  | maxEigenvalue | **0.0007** | **0.0007** | 0.0019 |
|  | medWeight | **0.0002** | **0.0003** | 0.0053 |
|  | transitivity | **0.0004** | **0.0005** | 0.0108 |
| DS2 | avgWeight | **0.0066** | **0.0019** | 0.0025 |
|  | avgClusteringCoeff | **0.0069** | **0.0023** | 0.0038 |
|  | avgLocalEff | **0.0069** | **0.0022** | 0.0035 |
|  | maxEigenvalue | **0.0077** | **0.0036** | 0.0079 |
|  | medWeight | **0.0054** | **0.0023** | 0.0128 |
|  | transitivity | **0.0069** | **0.0023** | 0.0038 |
