# Supplementary material for: Neural network features distinguish chemosensory stimuli in Caenorhabditis elegans
Source: PLoS Comput Biol. 2021 Nov 9;17(11):e1009591. doi: 10.1371/journal.pcbi.1009591 (PMC8604368; doi:10.1371/journal.pcbi.1009591)
Supplement: S22 Table — Performance achieved by logistic regression classifier on a specific classification task–namely, correctly classify responses based on the graph features computed for the first pulse of a stimulus session given a certain time bin and NMI bin. The nested leave-one-out cross validation accuracy, the mean and standard deviation of the accuracies of a null distribution built using 100 permutations of the labels, and the corresponding p-value, or relative position of its accuracy in the null distribution, are all listed. We only used non-standardized graph features computed on the Whole Network. Values in red attained significantly above-chance accuracies, and those in bold red did so in Data Sets 1 and 2. Some tasks did not exceed chance (e.g., time bin 15 x NMI bin 0.1), and this is indicated by a dashed line to indicate that no permutation testing was conducted. Chance is 25%. (DOCX) [file pcbi.1009591.s036.docx]

| Time bin (seconds) | NMI bin | Accuracy (%) | Permutation score (%, mean±s.d.) | p-value |
| --- | --- | --- | --- | --- |
| 15 | 0.05 | 33 | 18±10 | 0.1089 |
|  | 0.1 | 25 | - | - |
|  | 0.2 | 25 | - | - |
| 23 | 0.05 | 42 | 17±11 | 0.0495 |
|  | 0.1 | 17 | - | - |
|  | 0.2 | 13 | - | - |
| 30 | **0.05** | **50** | **17±11** | **0.0099** |
|  | 0.1 | 25 | - | - |
|  | 0.2 | 13 | - | - |
