# Supplementary material for: Neural network features distinguish chemosensory stimuli in Caenorhabditis elegans
Source: PLoS Comput Biol. 2021 Nov 9;17(11):e1009591. doi: 10.1371/journal.pcbi.1009591 (PMC8604368; doi:10.1371/journal.pcbi.1009591)
Supplement: S28 Table — B, D, I, N, and S refer to benzaldehyde, diacetyl, isoamyl alcohol, 2-nonanone, and NaCl (salt). The weights are log odds; hence, an unit increase in that row’s feature increases or decreases the log odds of the model predicting that the sample belongs to that column’s stimulus. The prefixes ‘Graph’ and ‘Activity’ indicate whether the feature is a graph or activity feature. The features with the largest positive and negative weights for each stimulus are colored red, and those found to be significantly modulated by stimulus identity via our mixed-effects model are highlighted. The features were standardized as this gave the best performance for the classifier (see S9 Table). (DOCX) [file pcbi.1009591.s042.docx]

| Feature | B | I | N | S |
| --- | --- | --- | --- | --- |
| Graph_degDist | -0.04±0.06 | 0.08±0.14 | -0.08±0.06 | 0.04±0.08 |
| Graph_shortestPaths | -0.15±0.16 | 0.63±0.28 | -0.39±0.17 | -0.08±0.07 |
| Graph_clusteringCoeff | -0.35±0.16 | 0.14±0.11 | -0.05±0.04 | 0.27±0.09 |
| Graph_localEff | -0.34±0.16 | 0.11±0.09 | -0.04±0.04 | 0.28±0.09 |
| Graph_participationCoeff | 0.25±0.26 | -0.5±0.23 | 1.26±0.47 | -1.01±0.5 |
| Graph_betweenCentrality | -0.04±0.11 | 0.13±0.12 | 0.48±0.25 | -0.58±0.25 |
| Graph_averageNeighborDeg | -0.25±0.12 | 0.09±0.07 | -0.13±0.07 | 0.29±0.11 |
| Graph_eigens | -0.64±0.29 | 0.05±0.05 | 0.36±0.16 | 0.23±0.13 |
| Graph_radius | -0.06±0.17 | -0.75±0.45 | -0.58±0.27 | 1.39±0.71 |
| Graph_diameter | -0.94±0.51 | 0.96±0.44 | -0.63±0.26 | 0.62±0.32 |
| Graph_modularity | -1.61±0.8 | 0.22±0.23 | 0.39±0.18 | 1.0±0.46 |
| Graph_numberOfModules | -0.24±0.22 | 0.07±0.14 | -0.3±0.2 | 0.48±0.36 |
| Graph_globalEff | -0.11±0.07 | -0.18±0.09 | 0.22±0.11 | 0.07±0.05 |
| Graph_assortCoeff | -0.01±0.05 | -0.04±0.12 | 0.1±0.07 | -0.04±0.08 |
| Graph_transitivity | -0.35±0.16 | 0.14±0.11 | -0.05±0.04 | 0.27±0.09 |
| Graph_maxEigenvalue | -0.29±0.14 | 0.12±0.08 | -0.12±0.06 | 0.3±0.11 |
| Graph_numberNodes | -0.04±0.06 | 0.08±0.14 | -0.09±0.06 | 0.04±0.08 |
| Graph_numberEdges | -0.06±0.07 | 0.1±0.15 | -0.08±0.06 | 0.04±0.08 |
| Graph_numComponents | -1.31±0.65 | 1.07±0.62 | 0.42±0.2 | -0.18±0.17 |
| Graph_averageWeight | -0.32±0.14 | 0.06±0.06 | -0.04±0.04 | 0.3±0.11 |
| Graph_medianWeight | -0.33±0.14 | -0.08±0.04 | -0.14±0.09 | 0.55±0.23 |
| Graph_eigenvectorCentrality | -0.26±0.21 | -0.38±0.14 | 0.88±0.33 | -0.25±0.2 |
| Activity_meanActivity | -0.17±0.19 | -0.04±0.08 | -0.23±0.21 | 0.44±0.19 |
| Activity_stdActivity | 0.56±0.34 | -0.92±0.51 | -0.03±0.2 | 0.39±0.23 |
| Activity_skewActivity | -0.55±0.24 | 0.4±0.17 | -0.47±0.23 | 0.62±0.26 |
| Activity_kurtActivity | 0.24±0.28 | -0.77±0.4 | 0.97±0.45 | -0.45±0.27 |
| Activity_power1 | -0.3±0.19 | -0.99±0.55 | 1.37±0.62 | -0.08±0.09 |
| Activity_power2 | 0.61±0.41 | -0.48±0.37 | -0.05±0.1 | -0.08±0.09 |
| Activity_power3 | -0.91±0.47 | 0.64±0.35 | 0.92±0.43 | -0.65±0.35 |
| Activity_peakFreq | -1.1±0.53 | 1.48±0.74 | 0.24±0.21 | -0.62±0.35 |
| Activity_meanFreq | 1.04±0.53 | -0.37±0.24 | -0.08±0.15 | -0.58±0.29 |
| Activity_stdFreq | -0.78±0.42 | -1.35±0.58 | 1.44±0.65 | 0.69±0.47 |
